# Modulated structure determination and ion transport mechanism of oxide-ion conductor CeNbO_4+δ_

**DOI:** 10.1038/s41467-020-18481-x

**Published:** 2020-09-21

**Authors:** Jian Li, Fengjuan Pan, Shipeng Geng, Cong Lin, Lukas Palatinus, Mathieu Allix, Xiaojun Kuang, Jianhua Lin, Junliang Sun

**Affiliations:** 1 College of Chemistry and Molecular Engineering, Peking University, 100871 Beijing, P. R. China; 2 Department of Materials and Environmental Chemistry, Stockholm University, 10691 Stockholm, Sweden; 3 Guangxi Key Laboratory of Optical and Electronic Materials and Devices, College of Materials Science and Engineering, Guilin University of Technology, 541004 Guilin, P. R. China; 4 School of Advanced Materials, Shenzhen Graduate School, Peking University, 518055 Shenzhen, P. R. China; 5 Institute of Physics, Academy of Sciences of the Czech Republic, v.v.i., Na Slovance 2, 182 21 Prague, Czech Republic; 6 CNRS, CEMHTI UPR3079, Univ. Orléans, 45071 Orléans, France

**Keywords:** Chemistry, Inorganic chemistry, Solid-state chemistry, Materials chemistry, Electronic materials

## Abstract

CeNbO_4+δ_, a family of oxygen hyperstoichiometry materials with varying oxygen content (CeNbO_4_, CeNbO_4.08_, CeNbO_4.25_, CeNbO_4.33_) that shows mixed electronic and oxide ionic conduction, has been known for four decades. However, the oxide ionic transport mechanism has remained unclear due to the unknown atomic structures of CeNbO_4.08_ and CeNbO_4.33_. Here, we report the complex (3 + 1)D incommensurately modulated structure of CeNbO_4.08_, and the supercell structure of CeNbO_4.33_ from single nanocrystals by using a three-dimensional electron diffraction technique. Two oxide ion migration events are identified in CeNbO_4.08_ and CeNbO_4.25_ by molecular dynamics simulations, which was a synergic-cooperation knock-on mechanism involving continuous breaking and reformation of Nb_2_O_9_ units. However, the excess oxygen in CeNbO_4.33_ hardly migrates because of the high concentration and the ordered distribution of the excess oxide ions. The relationship between the structure and oxide ion migration for the whole series of CeNbO_4+*δ*_ compounds elucidated here provides a direction for the performance optimization of these compounds.

## Abstract (web summary)

Materials with oxygen hyperstoichiometry received great attention in solid oxide fuel cells field because of the low activation energy of interstitial ion migration. Here the authors revealed the relationship between the structure and oxide ion migration for the whole series of CeNbO4+δ compounds.

## Introduction

Materials with oxygen hyperstoichiometry have excellent electronic, magnetic, and oxygen storage properties and can be used in a wide variety of applications^1–5^. For example, La_2_CuO_4+*δ*_ forms two phases depending on the oxygen content^6^: the phase with *δ* = 0 is semiconducting, while the other has a wide range of values (0.03 < *δ* < 0.18) and it becomes superconducting for *δ* = 0.08 with *T*_c_ ~ 38 K. The discovery of the series of 114 cobaltites ((Ln,Ca)_1_BaCo_4_O_7_) revealed the existence of closely related structures with various crystallographic symmetries and the possibility of oxygen nonstoichiometry in the range “O_7_”–“O_8.5_” in those systems, which opened up a new field for the investigation of strongly correlated electron systems. This change of oxygen stoichiometry, which induces the variation of the Co^2+^:Co^3+^ ratio in the system, is expected to influence the physical properties of these compounds considerably. This is the case of the oxygen-rich “114” cobaltites YBaCo_4_O_8_ and YbBaCo_4_O_7.2_, which were shown to be magnetically frustrated rather than magnetically ordered at low temperatures^7,8^. Therefore, oxygen hyperstoichiometry materials with useful functionalities are attractive subjects of research for chemists, physicists, or materials scientists.

In recent years, materials with oxygen hyperstoichiometry received great attention in the field of solid oxide fuel cells because of the low activation energy (*E*_a_) of interstitial ion migration. Well known and studied examples are apatites La_10-*δ*_(MO_4_)_6_O_3−1.5*δ*_ (M = Si, Ge)^9^, melilite La_1+*δ*_Sr_1-*δ*_Ga_3_O_7+0.5*δ*_^10^, layered perovskites La_2_NiO_4+*δ*_^11^, fluorite UO_2+*δ*_^12^, or scheelite La_0.2_Pb_0.8_WO_4+*δ*_^13^, Bi_1−*δ*_Sr_*δ*_VO_4−0.5*δ*_^14^, and also the subject of this study, CeNbO_4+*δ*_. CeNbO_4+*δ*_ is a mixed ionic and p-type electronic conductor with fast oxygen ion diffusion at moderate temperatures (total conductivity up to 0.030 S cm^−1^ at 850 °C; ion transference number up to 0.4; diffusion coefficient up to 8.3 × 10^−8^ cm^2^ s^−1^), making it a promising material for applications in energy generation and storage devices^15–23^. CeNbO_4+*δ*_ was first reported by Cava et al.^15^ in 1970s. It has been identified as a family of compounds with variable oxygen contents, with distinct phases CeNbO_4_, CeNbO_4.08_, CeNbO_4.25_, and CeNbO_4.33_. Although Thompson et al.^16^ successfully indexed the unit cell of these four compounds by selected area electron diffraction (SAED) in 1999, no progress on the structure solution of these phases was made until in 2016 Pramana et al.^17^ solved the structure of CeNbO_4.25_ by single-crystal X-ray diffraction (SCXRD) and revealed by molecular dynamics (MD) simulations that the fast ion migration occurs within planes of the neighboring NbO_*n*_ polyhedra. However, the atomic structures of CeNbO_4.08_ and CeNbO_4.33_ remained unknown, which hindered the full understanding of the oxide ion conduction behavior for the whole system of CeNbO_4+*δ*_.

In order to better understand the oxygen transport mechanism and to optimize its performance, atomic structures of CeNbO_4.08_ and CeNbO_4.33_ need to be understood. However, for CeNbO_4.08_ and CeNbO_4.33_, it was very difficult to grow large single crystal due to their specific syntheses. Furthermore, CeNbO_4.08_ appeared to be a (3 + 2)-dimensional incommensurately modulated structure with monoclinic symmetry and CeNbO_4.33_ a commensurately modulated structure with triclinic symmetry according to SAED^16^. The large unit cell parameters and complex diffraction patterns make it very difficult to determine their atomic structures by conventional powder X-ray diffraction (PXRD). Fortunately, nanocrystals and microcrystals can be treated as single crystals in electron microscopy. The recently developed 3D ED technique, continuous rotation ED^24–27^, can use single nanocrystals to obtain single-crystal diffraction data that can be used for structure determination by utilizing the software developed for SCXRD (ShelxT^28^, Superflip^29,30^).

In this study, we determine the incommensurately modulated structure of CeNbO_4.08_ and the superstructure of CeNbO_4.33_ by combining 3D ED, synchrotron X-ray powder diffraction (SPD), and neutron powder diffraction (NPD). Using the same methods, the superstructure of CeNbO_4.25_ was also re-determined. The structure models are sufficiently accurate to reveal the interstitial oxygen sites and allow to elucidate how the extra oxygen atoms change the structural connectivity. Combining the structural information with MD simulations, we describe the oxide ion migration mechanisms in CeNbO_4+*δ*_. The relationship between the structure and oxide ion migration for the whole series of CeNbO_4+*δ*_ compounds here provides means to optimize the performance of these compounds and to develop better oxygen hyperstoichiometric materials for a wide variety of applications.

## Results

**Fig. 1:**
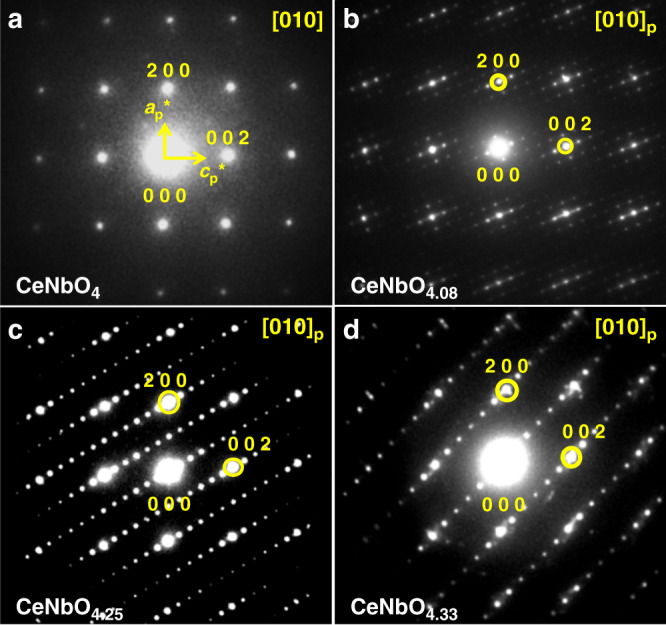
[010]_p_ zone axis SAED of CeNbO_4+*δ*_. **a** CeNbO_4.0_. **b** CeNbO_4.08_. **c** CeNbO_4.25_. **d** CeNbO_4.33_. The strong reflections in **b**–**d** have the same pseudo-tetragonal symmetry as the reflections of the parent structure in **a**. (p denotes parent material CeNbO_4_).

A series of phases of CeNbO_4+*δ*_ was obtained from CeO_2_ and Nb_2_O_5_ by the solid-state synthetic route in Supplementary Fig. 1. The most striking chemical feature of the studied cerium niobate phases is that they are progressively oxidized from one structure to another under relatively mild conditions. The oxygen contents of the phases were analyzed by thermogravimetric analysis (TGA), which yields formulae CeNbO_4.11(2)_, CeNbO_4.24(2)_, and CeNbO_4.33(1)_ (Supplementary Fig. 2). Figure 1 shows the [010]_p_ (p denotes parent material CeNbO_4_) zone axis SAED patterns of the CeNbO_4+*δ*_ (*δ* = 0, 0.08, 0.25, 0.33) phases. In all the four SAED patterns, the strong reflections have the same pseudo-tetragonal symmetry as that of the parent structure. However, for CeNbO_4.08_, CeNbO_4.25_, and CeNbO_4.33_, many additional reflections can be observed.

### Incommensurately modulated structure of CeNbO_4.08_

In previous transmission electron microscopic study, CeNbO_4.08_ was identified as a monoclinic (3 + 2)D incommensurately modulated phase with average unit cell parameters close to the parent CeNbO_4_ and modulation vectors q1 ~ (0.138, 0, 0.344), q2 ~ (0.345, 0, −0.138) and centering *I* = (½, ½, ½, 0, 0) (Supplementary Fig. 3a)^16^. In the present study, analysis of the [010]_p_ zone axis SAED of CeNbO_4.08_ indicated that the structure can be described as monoclinic (3 + 1)D incommensurately modulated with a single modulation vector *q* = 0.069a* + 0.175c* and a nonstandard centering *X* = (½, ½, ½, ½). However, satellites up to order 5 or even 7 are needed to describe the whole pattern (Supplementary Fig. 3b). The qualities of the LeBail fit of SPD are similar for the 2D modulation and 1D modulation, which further confirmed that the 1D modulation is the correct description (Supplementary Fig. 4). It is extremely unlikely that a single modulation vector would be able to describe two independent modulation vectors just by coincidence with such accuracy. Satellites up to order 7 in the synchrotron powder data are visible. However, the number of satellites is very large and the sensitivity of the SPD pattern to oxygen positions is relatively low, making it extremely challenging to solve the (3 + 1)D incommensurately modulated structure of CeNbO_4.08_ ab initio from the SPD pattern. Therefore, the 3D ED technique was applied to solve its incommensurately modulated structure.

**Fig. 2:**
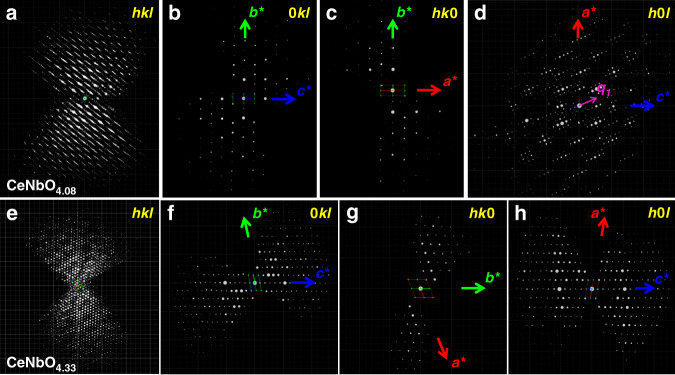
3D ED data of CeNbO_4.08_ and CeNbO_4.33_. 3D reciprocal lattice reconstructed from the 3D ED data of CeNbO_4.08_ (**a**) and CeNbO_4.33_ (**e**). **b** (*0kl*), **c** (*hk0*), and **d** (*h0l*) slices extracted from the reconstructed reciprocal lattice of CeNbO_4.08_, reflection conditions: 0*klm*: *k* + *l* = 2*n*. *hk0m*: *h* + *k* = 2*n*, *h0lm*: *h* + *m* = 2*n*, *l* + *m* = 2*n*, *h00m*: *h* + *m* = 2*n*, 0*k00*: *k* = 2*n*, 00 *lm*: *l* + *m* = 2*n*. **f** (*0kl*), **g** (*hk0*), and **h** (*h0l*) slices cut from the reconstructed reciprocal lattice of CeNbO_4.33_, reflection conditions: no special systematic reflections appear forbidden.

**Fig. 3:**
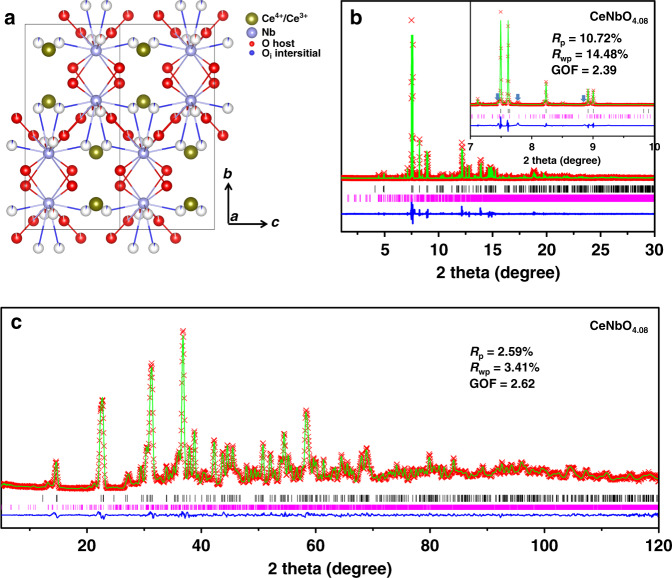
Structure characterization and analysis of CeNbO_4.08_. **a** Average structure of the (3 + 1)D incommensurately modulated structure of CeNbO_4.08_ along the [100]_p_ direction. The interstitial O3 (blue atom) is located between Ce cations within the Ce cationic chain. **b** Final Rietveld refinement against the SPD data for CeNbO_4.08_ with (3 + 1)D superspace. Note: the unexplained peaks at 7.46, 7.77, and 8.86 degree (marked at inset pattern) belong to the impurity phase of CeNbO_4.25_. **c** Final Rietveld refinement against the NPD data for CeNbO_4.08_ with (3 + 1)D superspace. (Red multi (×) symbol: observed profile, green curve: simulated profile, blue curve: difference profile, black Bragg line: main reflections, pink Bragg line: satellite reflections).

Due to the limitation of processing continuous mode 3D ED data with (3 + 1)D superspace, we processed the 3D ED data with approximate supercell. The 3D reciprocal lattice reconstructed from the 3D ED data of CeNbO_4.08_ are shown in Fig. 2a–d. Reciprocal lattice along the [010]_p_ direction can be cut from the reconstructed 3D reciprocal lattice (Supplementary Fig. 6b), in which the strong reflections show the similar pseudo-tetragonal symmetry as the [010]_p_ zone axis SAED of CeNbO_4._. Then we transformed the processed 3D ED data (hkl file based on the approximate supercell) to (3 + 1)D superspace. Reflection conditions indicated the superspace group of *X2/c(α0γ)0s* and satellites up to order 7 were required. The determination of the modulation functions turned out to be extremely difficult due to their complexity and large number of contributing harmonic waves. Finally, the (3 + 1)D model was constructed from the supercell structure. The structure contains one additional oxygen site (O3) compared with the parent structure of CeNbO_4_ (Fig. 3a, see Supplementary Information for details on the localization of this additional atoms). Detailed analysis of the interatomic distances and difference potential maps showed that the atom O1 has two positions—one is the main atomic domain while the other is occupied only when O1 coexists with O3. The modulation structure model was first refined against 3D ED with modulation functions up to order 7 and modulation of ADP parameters up to order 3 for Ce and 2 for Nb. The final modulated structure model of CeNbO_4.08_ was obtained by the Rietveld refinement of combined SPD data (Fig. 3b) and NPD data (Fig. 3c). Modulation functions up to order 7 were used together with isotropic atom displacement parameters. The model resulted in chemically reasonable bond distances (Supplementary Fig. 9) and modulation functions similar to the model obtained against 3D ED data (Supplementary Figs. 10 and 11).

### Supercell structure of CeNbO_4.33_

From the [010]_p_ zone axis SAED, the satellites in CeNbO_4.25_ and CeNbO_4.33_ can be indexed with a single vector *q* = 1/12 [204]_p_ and 1/3 [101]_p_, respectively. Thus the commensurately modulated structure of CeNbO_4.25_ and CeNbO_4.33_ can be described by supercell models. Again, 3D ED data were collected on CeNbO_4.33_ (Fig. 2e–h) and CeNbO_4.25_ (Supplementary Fig. 5e–h)_._ The reflection conditions derived from the 3D ED datasets indicated that the possible space group of CeNbO_4.33_ is *P*$\bar 1$. All the crystallographically unique Ce and Nb atoms as well as a part of oxygen atoms in the structure were located directly by using the software *SHELXT*^28^. The supercell structure model of CeNbO_4.25_ could also be obtained with 3D ED data. The final supercell structure model for CeNbO_4.33_ and CeNbO_4.25_ with interstitial oxygen sites were obtained by the Rietveld refinement of combined SPD and NPD data (Supplementary Figs. 12–15).

### Identification of the interstitial sites O_i_ in the structure models

**Fig. 4:**
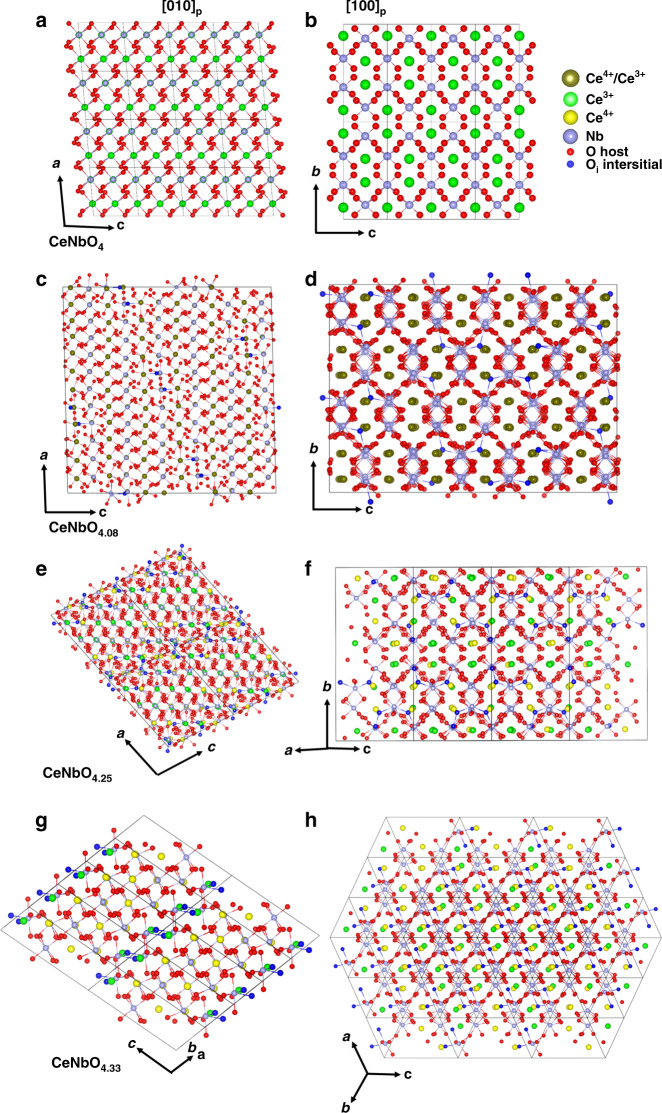
Structure model of CeNbO_4+*δ*_. CeNbO_4_ (**a**, **b**), oxidized CeNbO_4.08_ (**c**, **d**), CeNbO_4.25_ (**e**, **f**), and CeNbO_4.33_ (**g**, **h**) projected along the principal axes of [010]_p_ and [100]_p_. Displacement of oxygen from the original position in the parent structure is observed. Note that **c** and **d** were 6*a* × 2*b* × 6*c* approximant superstructure in (3 + 1)D incommensurately modulated model along [010]p and [100]p.

Comparing the three oxide supercell structures with the parent structure (Fig. 4 and Supplementary Fig. 16), the additional oxygen atoms incorporated into the CeNbO_4_ host lattice have relaxed the original oxide ion positions, which resulted in three different incommensurately or commensurately modulated structures. Figure 4 shows the structures of the parent CeNbO_4_ and the structures of CeNbO_4.08_, CeNbO_4.25_, and CeNbO_4.33_ projected along the principal axes of [010]_p_ and [100]_p_. Significant displacement of oxygen atoms from the original positions in the parent structure is observed and additional oxygen sites appear in the cationic layers, close to the interstitial position O3 identified in the average structure of CeNbO_4.08_ (Fig. 3a).

In the oxidized supercell structures, the excess oxygen atoms are located at one ((3 + 1)D incommensurately modulated model of CeNbO_4.08_), three (CeNbO_4.25_), and one (CeNbO_4.33_) fully occupied general Wyckoff sites, respectively. The interstitial sites are the blue atoms in Fig. 4 (O3 in CeNbO_4.08_ 6*a* × 2*b* × 6*c* approximant superstructure structure, O4, O11, and O41 in CeNbO_4.25_ and O13 in CeNbO_4.33_). Bond valence sum (Supplementary Tables 2 and 3 and Supplementary Fig. 17) analysis shows that some of the Ce positions are Ce^4+^ sites. The change of the oxidation state of Ce is responsible for the shorter Ce-O bond lengths and allows that excess oxygen is incorporated into the host lattice at the interstitial sites between Ce cations (Fig. 4c–h). In CeNbO_4.33_, the amount of additional oxygen is so much that part of the oxygen atoms are pushed away from the original normal site into an interstitial site (O11) due to the relaxation of the structure. The interstitial site oxygens (O11, O13) between Ce cations are occupied in an ordered manner along the *a* and *c* axes in CeNbO_4.33_ (Supplementary Fig. 18). The ordered interstitial oxygen (O13) and the relaxation of the other oxygen site (O11) generate a zigzag-shaped [Nb_6_O_26_]_∞_ infinite chain comprising edge-sharing [NbO_6_]_4_ units bridged by edge-sharing [NbO_6_]_2_ units through vertex (Supplementary Fig. 18a).

### Oxide ion migration

**Fig. 5:**
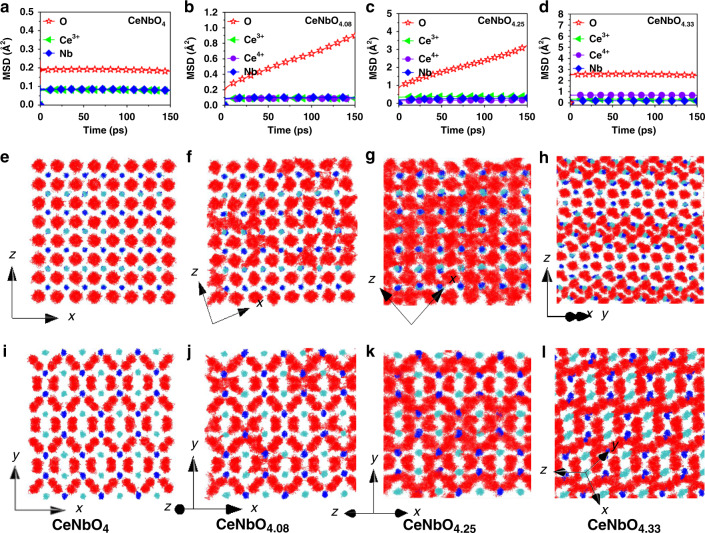
Molecular dynamics simulations study of CeNbO_4+*δ*_. Calculated MSD values of Ce^3+/4+^, Nb, and O atoms as a function of simulation time for CeNbO_4_ (**a**), CeNbO_4.08_ (**b**), CeNbO_4.25_ (**c**), and CeNbO_4.33_ (**d**) from the MD simulation at 1200 °C. Trajectory scatter plots for CeNbO_4_ (**e**, **i**), CeNbO_4.08_ (**f**, **j**), CeNbO_4.25_ (**g**, **k**), and CeNbO_4.33_ (**h**, **l**) at 1200 °C where cyan, blue, and red dots represent Ce^3+/4+^, Nb^5+^, and O^2−^ atoms, respectively.

MD simulations based on interatomic potential method were performed to elucidate the dependence of oxide ion migration on the variation of oxygen hyperstoichiometry and structure in CeNbO_4+*δ*_ compounds. The mean square displacement (MSD) values (Fig. 5a–d and Supplementary Fig. 19) and scatter plots (Fig. 5e–l and Supplementary Fig. 20) indicate that all atoms in the parent CeNbO_4_ show only lattice vibration without long-range migration as expected. With inclusion of the excess oxygen into the host lattice of CeNbO_4_, the oxide ions become mobile in CeNbO_4.08_ and CeNbO_4.25_ but hardly migrate in CeNbO_4.33_. The MD simulations show that CeNbO_4.08_ and CeNbO_4.25_ phases have similar oxide ion migration pathways.

**Fig. 6:**
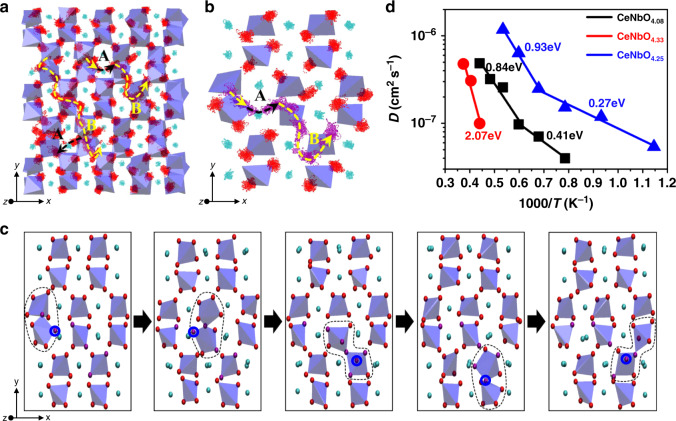
The oxide ionic transport mechanism of CeNbO_4+*δ*_. **a**, **b** Trajectory scatter plots of Ce and O atoms showing the migration paths A (black dash dot line) and B (yellow dash dot line) with the NbO_*n*_ polyhedral network embedded in, and **c** snapshots of long-range oxide ion (marked with the blue circle) migration involving the breaking and reforming of the Nb_2_O_9_ units (highlighted by dashed lines) in CeNbO_4.08_ from the MD simulations at 1200 °C. In order to illustrate the Nb_2_O_9_ breaking and reforming mechanism clearly, in **b**, **c** the weak Nb-O bonds >2.2 Å are not included in the NbO_*n*_ polyhedral units. **d** Arrhenius plot of the oxygen diffusion coefficient calculated using the MSD values of oxygen atoms for CeNbO_4+*δ*_ (*δ* = 0.08, 0.25, 0.33). The activation energies are labeled.

Two kinds of oxide ion migration events contributing to the long-range migration can be identified in the MD simulations of CeNbO_4.08_ (Fig. 6a, b). The first one is oxide ion migration between NbO_*n*_ polyhedra isolated by the Ce cationic chain by ~5.0–5.4 Å (labeled as path A). In this migration event, the oxide ion leaves one NbO_*n*_ polyhedron and passes through the interstitial sites (referred to as O_i_) within the chain of Ce cations along the *a* or *c* axis of the parent CeNbO_4_ structure to subsequently enter into the coordination environment of another NbO_*n*_ polyhedron located at the other side of the Ce chain through a knock-on process. The second type of migration is the oxide ion migration between the neighboring NbO_*n*_ polyhedra separated by ~3.6−4.2 Å (labeled as path B) through a chain knock-on process via the NbO_*n*_ polyhedral rotation and deformation. This migration event also frequently involves the interstitial sites O_i_ (Fig. 6a, b). MD simulations indicate that the migration along path A is much less frequent than that along path B, which is consistent with the larger separation between the NbO_*n*_ polyhedral units involved in path A than that in path B.

As Nb atoms are usually found in 6 or 4 coordinate environments and the Nb–O bond lengths normally vary within 1.8–2.2 Å, the Nb–O bonds >2.2 Å were not included in the NbO_*n*_ polyhedral units during a close examination of oxide ion migration, which simplifies the description of the polyhedral units and assists understanding the complex migration process. This representation leads to isolated tetrahedral NbO_4_ units and corner-sharing Nb_2_O_9_ units most of the time during simulation. The oxide ions were found to move between the polyhedral units in the long-range migration routes composed of paths A and B mainly through the synergic-cooperation mechanism involving the continuous breaking and reformation of the Nb_2_O_9_ units assisted by rotation and deformation of the NbO_*n*_ polyhedra and a knock-on process between the oxygen atoms (Fig. 6b, c and Supplementary Video 1). Therefore, the interstitial sites within the Ce cationic chain and NbO_*n*_ polyhedra define more like 3D pathways for the oxide ion migration in CeNbO_4.08_, which is consistent with the fact that inclusion of the extra oxygen atoms disturbs anisotropically the polyhedral linkage in CeNbO_4_.

The same oxide ion migration pathways are found also in CeNbO_4.25_ phase in our study. Oxide migration in CeNbO_4.25_ was analyzed also by Pramana et al.^17^. They identified anisotropic oxide ion diffusion paths within the linked NbO_6_ layers and pointed out that migration between the adjacent layers also took place when the migration paths along the linked NbO_6_ layers were blocked. The migration routes proposed by Pramana et al.^17^ are similar to those composed of paths B only identified in our study. However, the work of Pramana et al.^17^ did not identify the interstitial sites and as a consequence did not recognize migration paths going through the interstitial sites and the related dynamic process of the adaptation of the polyhedral units to the oxide ion migration. The proper description of the interstitial sites turns out to be crucial for the correct description of the full 3D long-range migration and also for the elucidation of the knock-on dynamic process of breaking and reformation of the Nb_2_O_9_ units during the oxide ion migration.

The calculated oxide diffusion coefficients of CeNbO_4.08_ are about one magnitude lower than that of CeNbO_4.25_ (Fig. 6d). This is ascribed to the larger concentration of the mobile charge carriers in CeNbO_4.25_. However, the oxide ions in CeNbO_4.33_ phase with more excess oxygen are essentially immobile at the same simulation temperatures compared with CeNbO_4.08_ and CeNbO_4.25_. The well-organized mixed corner/edge-sharing zigzag-shaped polyhedral [Nb_6_O_26_] chains in CeNbO_4.33_ phase resulting from the ordered distribution of the excess oxide ions have much less flexibility on the rotation and deformation. This rigidity blocks the transfer of the oxide ions between the polyhedral units. Also the high concentration of excess oxide ions in the oxygen sublattice seems to have a significant blocking effect to the oxide ion migration. The oxide ions in CeNbO_4.33_ phase get mobile only when the temperature is increased to above 2200 °C during the MD simulations (Supplementary Fig. 21). Even in that case, the migration path is confined only along the [Nb_6_O_26_] chain (Supplementary Fig. 22). The large *E*_a_ (~2.07 eV) calculated from the Arrhenius plot of oxygen diffusion coefficients confirm that the oxide ions are hardly mobile in CeNbO_4.33_ phase (Fig. 6d).

The MD simulations show an increase of *E*_a_ for the oxide ion migration with the temperature for both CeNbO_4.08_ and CeNbO_4.25_ phases (Fig. 6d), in accordance with the results by Pramana et al.^17^. The low-temperature *E*_a_ values (0.27–0.41 eV) are close to those from the conductivity data of CeNbO_4+*δ*_ (*δ* = 0.08, 0.25, 0.33; Supplementary Fig. 23), which did not show apparent *E*_a_ changes when placed under N_2_ flow and below 600 °C to maintain their oxygen contents (Supplementary Fig. 2). However, CeNbO_4+*δ*_ displays mixed electronic and oxide ion conduction and it is hard to quantify the electronic and ionic contributions to the conductivity. Therefore, the calculated *E*_a_ from the MD simulations at low temperatures here cannot be compared directly with the experimental values from these conductivity measurements. Owing to the instability of the CeNbO_4+*δ*_ phases to lose the extra oxygen at temperatures above 600 °C even in the O_2_-rich atmosphere^18,20^, there is no accurate *E*_a_ in higher temperature region for direct comparison with the calculated high-temperature *E*_a_ value for each phase. However, the high-temperature *E*_a_ values for CeNbO_4.08_ (0.84 eV) and CeNbO_4.25_ (0.93 eV) are close to the experimental *E*_a_ (~0.99 eV) for oxide diffusion in CeNbO_4+*δ*_ from the ^18^O tracer diffusion measurements by Packer et al.^22^, further validating our MD simulations. The increase of *E*_a_ with the temperature may be explained by the fact that the concentration of mobile oxide anions in CeNbO_4+*δ*_ could depend on the temperature. At low temperatures, the loosely bonded oxide ion could be the major mobile oxide ion, while at higher temperatures the more strongly bonded oxide ions start to move and contribute to the oxide ion migration, which requires extra energy and therefore increases the total *E*_a_.

In nonstoichiometric CeNbO_4+*δ*_, the incorporation of extra oxygen atoms is coupled by the oxidation of Ce^3+^ to smaller Ce^4+^ and the interstitial oxide ion migrations in the scheelite-based CeNbO_4.08_ and CeNbO_4.25_ take place through a synergic mechanism of breaking and reformation of the Nb_2_O_9_ dimers, which is possible thanks to the spatial proximity of the tetrahedral units. This is akin to the continuous breaking and reformation of the tetrahedral dimers in the vacancy-mediated oxide ion conductors based on isolated tetrahedral anion structures, e.g., La_1–*x*_Ba_1+*x*_GaO_4-0.5*x*_^31^ and Bi_1–x_Sr_x_VO_4–0.5*x*_^14^. However, in these two oxygen-vacancy conducting materials, the tetrahedral-dimer-assisted oxide ion migration does not modify the coordination number, while in the case of CeNbO_4+*δ*_ the coordination number in the polyhedral units involved in the oxide ion migration changes. The oxide ion migration mechanism in CeNbO_4+*δ*_ revealed here thus emphasizes the key roles of the coordination-number-variable cations as well as rotation and deformation flexibility of 4/5-coordinated polyhedral units for the oxide ion migration. This is generally consistent with the previous findings in interstitial oxide ion conducting apatite^31^ and melilite-type materials^10^. Therefore, the structures containing cations forming polyhedral units with rotation, deformation, and coordination flexibility, e.g., Ga, Ti, V, Nb, Mo, and W, are promising candidates for new oxide ion conductors if, at the same time, flexible oxidation state or donor substitution with smaller cations allows the introduction of excess oxide ions.

## Discussion

In summary, atomic structures of three oxygen hyperstoichiometric materials (CeNbO_4.08_, CeNbO_4.25_, CeNbO_4.33_) were determined by combining data from 3D ED, SPD, and NPD. The superstructure of CeNbO_4.33_ and the (3 + 1)D incommensurately modulated structure of CeNbO_4.08_ were obtained for the first time, to the best of our knowledge. The interstitial sites O_i_ were identified in all the three compounds and the structure analysis elucidates how the structure adapts for the oxygen hyperstoichiometry change, advancing our understanding of the complex CeNbO_4+*δ*_ system. Cationic size contraction of Ce upon the oxidation allows not only the incorporation of excess oxygen into the host lattice of CeNbO_4_ but also the relaxation of the NbO_*n*_ polyhedra and their interconnection through mixed corner/edge-sharing in three dimensions. MD simulations show that, with the inclusion of the excess oxygen into the host lattice of CeNbO_4_, the oxide ions become mobile in CeNbO_4.08_ and CeNbO_4.25_ with coordination-number-variable network but hardly migrate in the CeNbO_4.33_ phase owing to the ordered distribution of the excess oxide ions and the 6-coordinated [Nb_6_O_26_] polyhedral chain network with constrained deformation and rotation. Two kinds of oxide ion migration events are identified in CeNbO_4.08_ and CeNbO_4.25_ involving the interstitial O_i_ sites: (i) migration between the NbO_*n*_ polyhedra isolated by Ce cations; (ii) migration between neighboring NbO_*n*_ polyhedra. These two processes together form a long-range 3D network of migration pathways through which the oxygen ions migrate via a synergic-cooperation knock-on mechanism involving the continuous breaking and reformation of the Nb_2_O_9_ units assisted by the polyhedral rotation and deformation. The relationship between the structure and oxide ion migration for the whole series of CeNbO_4+*δ*_ compounds here provides means to optimize the performance of these compounds and to develop better oxygen hyperstoichiometric materials for a wide variety of applications.

## Methods

### Materials

The parent material CeNbO_4_ was prepared by traditional solid-state reaction. The powders of CeO_2_ and Nb_2_O_5_ were mixed and homogenized through grinding with an agate mortar and a pestle. The mixtures were annealed at 1623 K for 10 h and then sintered at 1073 K for 10 h in flowing argon gas atmosphere. The yellow colored powder, pure phase of CeNbO_4_, was finally obtained. The oxidized phases CeNbO_4.08_, CeNbO_4.25_, and CeNbO_4.33_ were synthesized as described in Supplementary Fig. 1. In short, the CeNbO_4.08_ phase was obtained by heating CeNbO_4_ in air at 1123 K for 15 min, then quenching in the furnace to 948 K, holding at 948 K for another 20 min, and finally quenching to room temperature in air. CeNbO_4.25_ was prepared by heating CeNbO_4_ at 873 K in air for 24 h and then cooling in furnace to room temperature. The CeNbO_4.33_ was prepared by heating CeNbO_4_ at 673 K in flowing oxygen atmosphere for 72 h and then cooling to room temperature under oxygen atmosphere. The powder was reground and then reheated under the same conditions until the powder was in a single crystalline phase (Schematic representation of the synthesis of CeNbO_4+*δ*_ is shown in Supplementary Fig. 1).

### Characterizations

3D ED data was collected on a 200 kV JEOL JEM-2100 transmission electron microscope. The goniometer was continuously rotated while SAED patterns were simultaneously captured from crystals with the sizes ranging from 100 to 500 nm using the quad hybrid pixel detector (Timepix). The 3D ED datasets were then processed using the X-ray Detector Software package^32^, which can export the results as an hkl list. TGA was performed on a TGA-Q500 from room temperature to 1000 °C with a heating rate of 10 °C min^−1^ under flowing N_2_ atmosphere. The AC impedance spectroscopy (IS) measurements were performed with a Solartron 1260 frequency response analyzer over the 10^−1^–10^−7^ Hz frequency range within the 100–700 °C temperature range under N_2_ atmosphere flows. Prior to the IS measurements, the platinum paste was coated on the opposite faces of the pellets to form electrodes. Conventional PXRD pattern for parent phase CeNbO_4_ was collected at room temperature on a PANalytical X’Pert Pro diffractometer in Debye–Scherrer geometry with Cu Kα1 radiation with a minimum full width half maximum of 0.028°. NPD data were collected on CeNbO_4+*δ*_ at ambient temperature over the 10–120° 2*θ* range at 2*θ* intervals of 0.05° on the 3T2 diffractometer at Laboratoire Leon Brillouin (France) using wavelength *λ* = 1.22997 Å (CeNbO_4.08_ and CeNbO_4.33_) and *λ* = 1.54 Å (CeNbO_4.25_). High-intensity and high-resolution SPD data were recorded on CeNbO_4+*δ*_ (*δ* = 0.08, 0.25 and 0.33) on the 11BM diffractometer at the Advanced Photon Source, Argonne National Laboratory. SPD data were collected over the 0.5–40° 2*θ* range with a 0.001° step size at room temperature with 0.3 mm sample capillary using *λ* = 0.4130370 Å. The structure models derived from the 3D ED data were used as initial models for Rietveld refinements by combining SPD with NPD using Jana 2006^33^ for (3 + 1)D incommensurately modulated structure of CeNbO_4.08_ and the software Topas Academic Version 5^34^ for CeNbO_4.25_ and CeNbO_4.33_.

### MD simulations

The oxide ion migration in CeNbO_4+*δ*_ was investigated through MD atomistic simulations based on interatomic potential approach with the DL_POLY code^35,36^. The Buckingham potential function^37^ was used to model interactions between ions and the shell model^38^ to describe the electronic polarizability. The interatomic potential parameters, which were used in the previous atomistic simulations of CeNbO_4_ and CeNbO_4.25_ by Pramana et al.^17^, were slightly modified (Supplementary Table 4), especially regarding the Ce^4+^-O^2−^ potential parameters^39^ for better reproduction of all experimental CeNbO_4+*δ*_ structures (Supplementary Table 5) by the General Utility Lattice Program (GULP)^39,40^. The lattice parameters and most of the bond lengths were reproduced within ±6% error except for 2–4 bond lengths in each oxidized phase showing relatively large discrepancies (±10–20%) from the experimental values. The MD simulations were performed for the whole series of CeNbO_4+*δ*_ using the updated interatomic potential parameters. The simulation box consisted of a 6 × 3 × 6 supercell containing 2592 atoms for the parent CeNbO_4_, a 1 × 3 × 1 supercell containing 2112 atoms for CeNbO_4.08_, a 3 × 2 × 4 supercell containing 7200 atoms for the CeNbO_4.25_, and a 5 × 5 × 3 supercell containing 2850 atoms for CeNbO_4.33_. The systems were equilibrated first under a constant pressure of 1 atm at specific temperatures within 1273–2673 K for 10^5^ time steps with a time step of 0.1 fs before carrying out the main MD simulation for 200 ps with 2 × 10^6^ time steps in the NVT ensemble. The Visual Molecular Dynamics package^41^ was used to perform MD data analysis and the MSDs were calculated with the nMoldyn3 code^42^. Oxygen diffusion coefficients were calculated from the slope of the MSD plots as a function of simulation time.

## Supplementary information

Supplementary Info

Supplementary Video_1

## Data Availability

All relevant data supporting the findings of this study are available from the corresponding authors upon request.
