# Supplementary material for: Modulated structure determination and ion transport mechanism of oxide-ion conductor CeNbO4+δ
Source: Nat Commun. 2020 Sep 21;11:4751. doi: 10.1038/s41467-020-18481-x (PMC7506534; doi:10.1038/s41467-020-18481-x)
Supplement: Supplementary file 1 — Supplementary Info [file 41467_2020_18481_MOESM1_ESM.docx]

**Supplementary Information**

**Modulated structure determination and ion transport mechanism of oxide-ion conductor CeNbO_4+δ_**

Li et al.

**This PDF file includes:**

Supplementary Note

Supplementary Figures 1-23

Supplementary Tables 1-5

Supplementary References

**Supplementary Note**

**3D ED data collection and processing**

The powder sample was crushed in an agate mortar, dispersed in absolute ethanol, and treated by ultrasonication for 5 minutes. Then, a droplet of suspension was transferred to a copper grid. The 3D ED data were collected on 200 kV JEOL JEM-2100 transmission electron microscope using the software instamatic^1^. During the data collection, the goniometer was rotated continuously while the selected area electron diffraction patterns were captured from the crystal simultaneously by a quad hybrid pixel detector (Timepix). Collected datasets were processed using X-ray Detector Software (XDS)^2^, which generated the hkl files that were used for structure solution and refinement. The 3D reciprocal lattice was reconstructed using the REDp software^3^.The detail of data collection and processing of CeNbO_4+δ_ are shown in Supplementary Table 1.

**Unit cell determination of CeNbO_4.08_, CeNbO_4.25_, and CeNbO_4.33_**

In the selected area electron diffraction (SAED) pattern of CeNbO_4.08_ along the [010]_p_ zone axis, the satellite reflections can be seen to run along the ~[2***a*_p_**^*^, 0***b*_p_**^*^, 5***c*_p_**^*^] and ~[5***a*_p_**^*^, 0***b*_p_**^*^, -2***c*_p_**^*^] directions of the reciprocal space (Supplementary Fig. 7b). Thus, the corresponding approximate supercell in reciprocal space of CeNbO_4.08_ was determined to

2***a*_r1_***~1/29[4, 0, 10]_p_*

2***c*_r1_***~1/29[10, 0, -4]_p_*

where p and r represent the parent and resultant phase, respectively.

[502]_p_/[001]_r1_ slice cut from the 3D ED reconstructed reciprocal lattice (Supplementary Fig. 8a) shows that ***b*_r1_*** = [010]_p_*. Thus, the resultant super cell in real space derived from the observed reciprocal space is given by a matrix:

$$\left( \begin{matrix} a_{r1} \\ b_{r1} \\ c_{r1} \end{matrix} \right)=\left( \begin{matrix} 2 & 0 & 5 \\ 0 & 1 & 0 \\ 5 & 0 & -2 \end{matrix} \right)\left( \begin{matrix} a_{p} \\ b_{p} \\ c_{p} \end{matrix} \right)$$

The approximate super cell of CeNbO_4.08_ that extracted from TEM was a monoclinic cell with *a* ~ 28.31Å, *b* ~ 11.49Å, *c* ~ 28.57Å, and *β* ~ 91.28º.

In the case of CeNbO_4.25_ phase (Supplementary Fig. 7c and Supplementary Fig. 8b), the resultant reciprocal space unit cell is given by ***a*_r2_*** = 1/12[204]_p_*, ***b*_r2_*** = 1/4[020]_p_*, and ***c*_r2_*** = 1/3[$10\bar{1}$]_p_*. Thus, the resultant superstructure real space unit cell derived from the observed reciprocal space is given by a matrix.

$$\left( \begin{matrix} a_{r2} \\ b_{r2} \\ c_{r2} \end{matrix} \right)=\left( \begin{matrix} 2 & 0 & 2 \\ 0 & 2 & 0 \\ 2 & 0 & -1 \end{matrix} \right)\left( \begin{matrix} a_{p} \\ b_{p} \\ c_{p} \end{matrix} \right)$$

The unit cell extracted from TEM was *a* ~ 14.38 Å, *b~* 22.79 Å, *c* ~ 11.83Å, and *β* ~105.10º.

For the case of CeNbO_4.33_ phase, the resultant supercell in reciprocal space can be determined as ***a*_r3_*** = [1$\bar{1}$0]*_p_-1/3[101]_p_* = 1/3[2$\bar{3}\bar{1}$]_p_*, ***b*_r3_*** = [110]_p_*-1/3[101]_p_* = 1/3[23$\bar{1}$]_p_*, and ***c*_r3_*** = 1/3[101]_p_* (Supplementary Fig. 7d and Supplementary Fig. 8c-d). Hence, the corresponding relationship in real space unit cell derived from the observed reciprocal space can be proposed by a matrix:

$$\left( \begin{matrix} a_{r3} \\ b_{r3} \\ c_{r3} \end{matrix} \right)=\left( \begin{matrix} 1/2 & -1/2 & -1/2 \\ 1/2 & 1/2 & -1/2 \\ 1 & 0 & 2 \end{matrix} \right)\left( \begin{matrix} a_{p} \\ b_{p} \\ c_{p} \end{matrix} \right)$$

Such unit-cell transformation leads to a triclinic cell: *a* ~ 6.72Å, *b* ~ 6.90Å, *c* ~ 11.27Å, *α* ~ 99.13º, *β* ~ 100.03º, and *γ* ~ 110.46º.

**Super cell structure solution of CeNbO_4.08_, CeNbO_4.25_, and CeNbO_4.33_**

The super cell structure of CeNbO_4.08_, CeNbO_4.25_, and CeNbO_4.33_ were solved with 3D ED by using the software *SHELX*T^4^ that developed for single crystal XRD. The similar structure model was also produced by Superflip^5^. 1 unique Ce and 1 unique Nb in CeNbO_4_, 15 unique Ce and 15 unique Nb in CeNbO_4.08_, 12 unique Ce and 12 unique Nb in CeNbO_4.25_, and 3 unique Ce and 3 unique Nb in CeNbO_4.33_ and part of oxygen atoms were located directly with the program Superflip. All the O atoms (2 unique O in CeNbO_4_, 59 unique O in CeNbO_4.08_, 51 unique O in CeNbO_4.25_, and 13 unique O in CeNbO_4.33_) were determined by the difference Fourier map.

**Locating the additional O3 atom in (3+1)D incommensurately modulated structure of CeNbO_4.08_**

Three datasets are available: 3D ED, synchrotron XRPD, and NPD. Each of them has their advantages and disadvantages:

- 3D ED: single crystal data, many satellites visible, but only kinematical refinement possible, limited sensitivity to oxygen positions.

- SPD: good refinement of unit cell and modulation vectors, satellites up to order 7 visible, high sensitivity to metal atoms but very low sensitivity to oxygen positions.

- NPD: broad reflections, but much better sensitivity to oxygen positions.

The position of O3 atom was located from difference maps. The xi-x4 difference Fourier maps (below) shows the position of the O3 atom from the NPD data, 3D ED data, and SPD data. The signals of the additional O3 from NPD data were quite clear and unambiguous but not very clear from SPD data. The final modulated model refinement was performed on combining SPD and NPD refinement.

The xi-x4 difference Fourier maps at the position of the O3 atom from the NPD data:


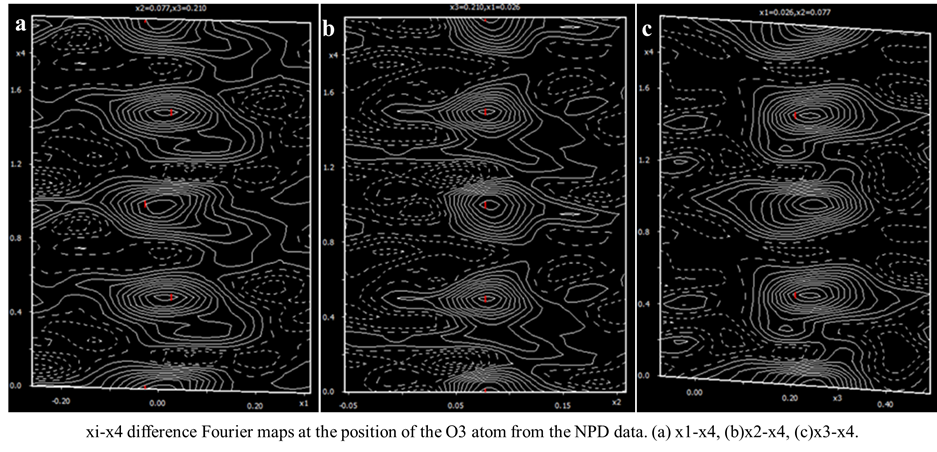


The short red lines are the existence domains of the O3 atoms. The length was set to correspond to the total of 0.08 oxygen atoms per formula unit in agreement with the composition. The signal is quite clear and unambiguous.

The xi-x4 difference Fourier maps at the position of the O3 atom from the 3D ED data:


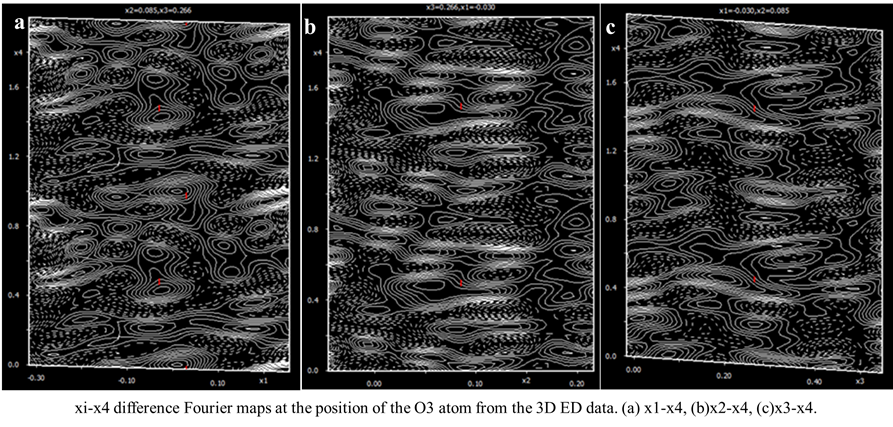


Here the signal is much less clear. However, there is a maximum near the expected position, which is (marginally) higher than the surrounding noise. Alone it could not be taken as an indication of the atom, but as a confirmation of the finding in NPD.

A similar observation can be made from the SPD data – the signal seems to be at the level of noise, but it is there (only x2-x4 section shown):

x2-x4 difference Fourier maps at the position of the O3 atom from the SPD data

**
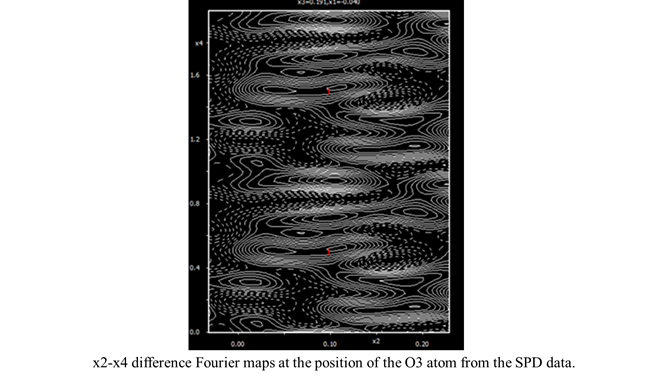
**

**Supplementary Figures**

**
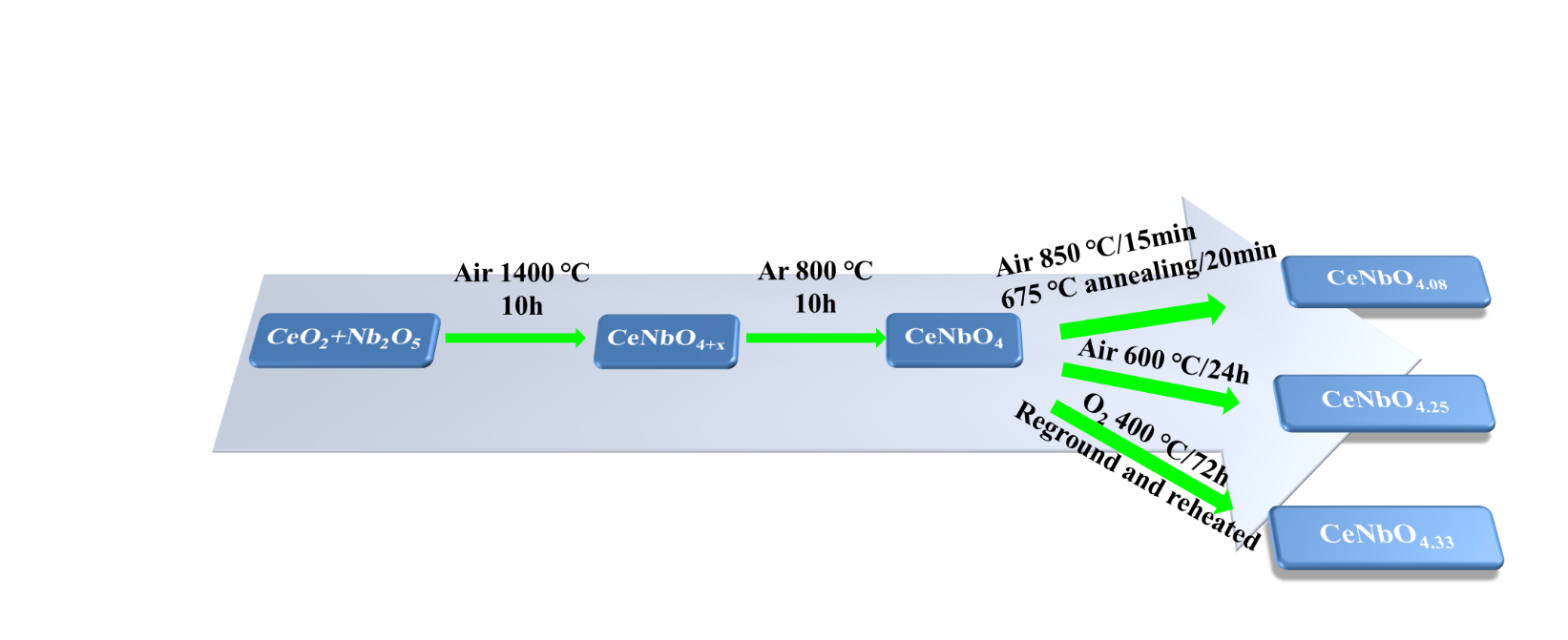
**

**Supplementary Fig. 1** Synthetic route of the three oxygen hyperstoichiometry materials.


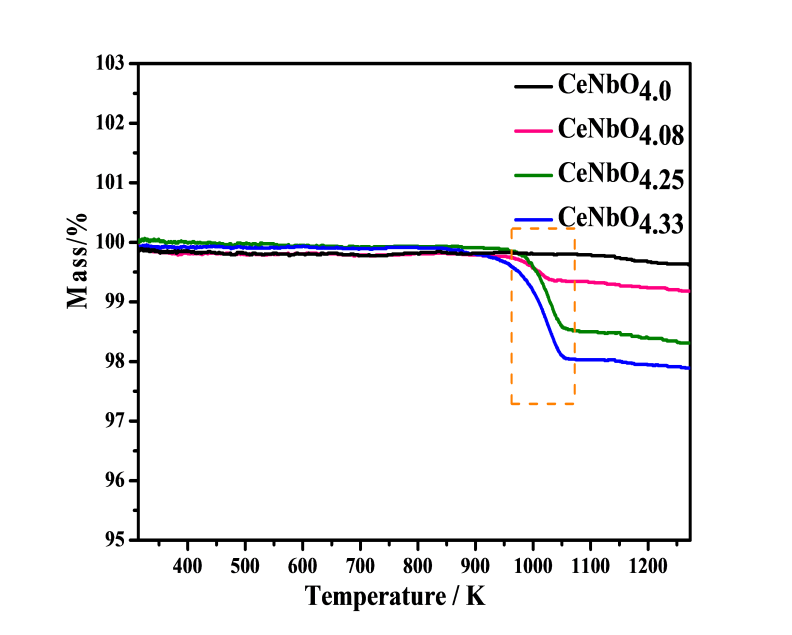


**Supplementary Fig. 2** Thermogravimetric analysis of CeNbO_4+δ_ under flowing N_2_ atmosphere. All the oxide material would be reduced to CeNbO_4_.


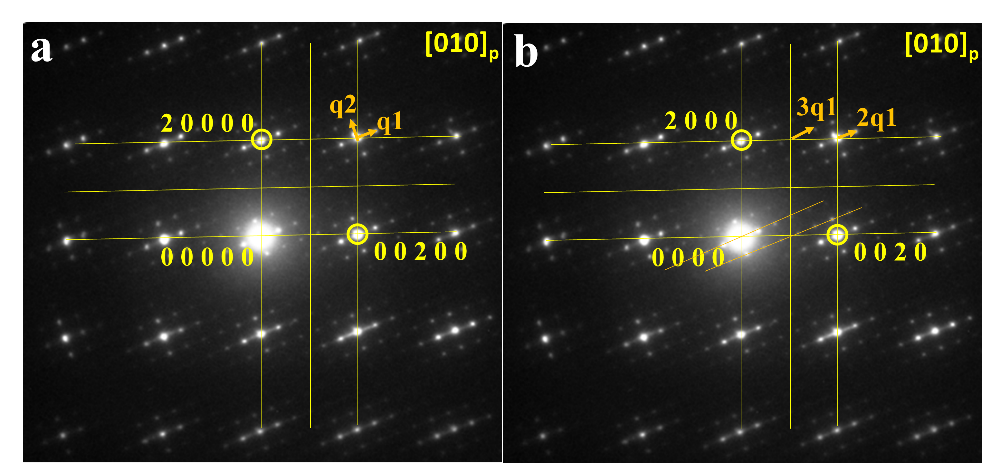


**Supplementary Fig. 3** [010]_p_ zone axis SAED of CeNbO_4.08_. Indexing as (3+2)D incommensurately modulated phase with modulation vectors q1~[0.138, 0, 0.344]*p and q2~[0.345, 0, -0.138]*p (p for parent) and centering I = (½, ½, ½, 0, 0) (**a**), and monoclinic (3+1)D incommensurately modulated with a single modulation vector q = 0.069a*+0.175c* and nonstandard centering X = (½ ½ ½ ½) (**b**).


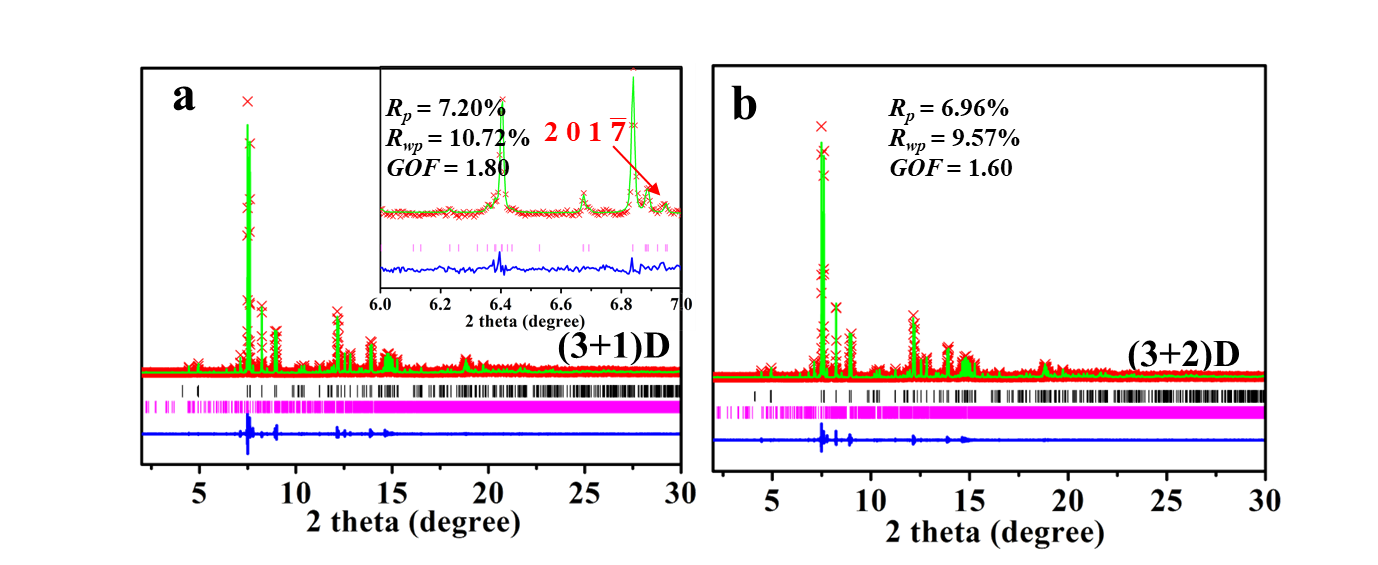


**Supplementary Fig. 4** LeBail fitting of CeNbO_4.08_ against SPD data with (3+1)D superspace symmetry (**a**) and (3+2)D superspace symmetry (**b**). (Red × symbol: observed profile, green curve: simulated profile, blue curve: difference profile, black Bragg line: main reflections, pink Bragg line: satellite reflections)


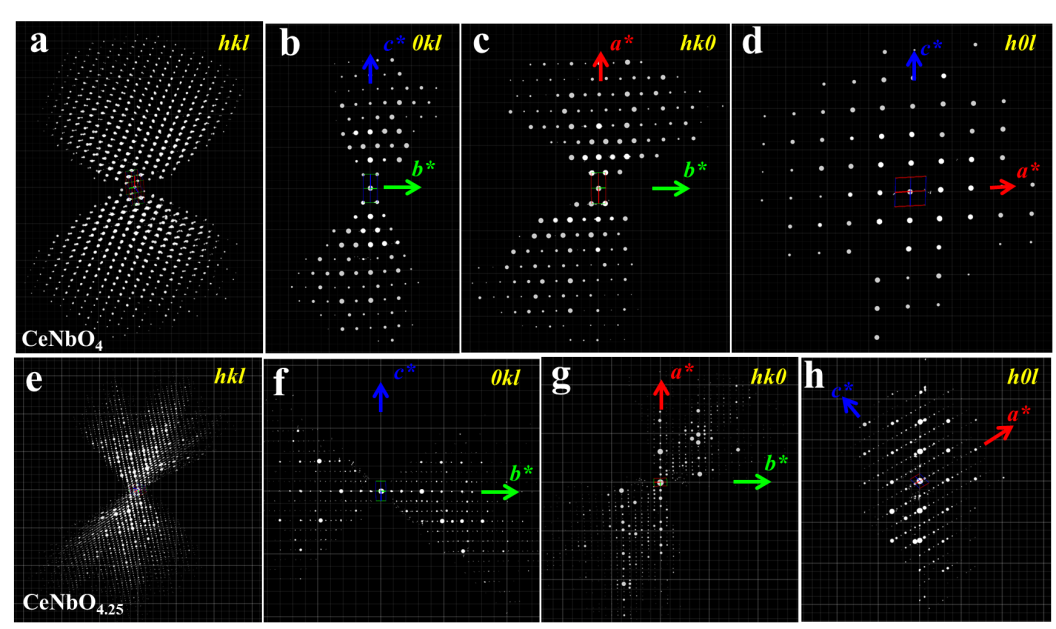


**Supplementary Fig. 5** 3D reciprocal lattice reconstructed from the 3D ED data of CeNbO_4_ (**a**), CeNbO_4.25_ (**e**). **b** (*0kl*), **c** (*hk0*), and **d** (*h0l*) slices extracted from the reconstructed reciprocal lattice of CeNbO_4_, reflection conditions: *hk0*: *h*+*k*=2n, *h0l*: *h*=2n, *l*=2n, *h00*: *h*=2n, 0*k0*: *k*=2n, 0*0l*: *l*=2n. **f** (*0kl*), **g** (*hk0*), and **h** (*h0l*) slices cut from the reconstructed reciprocal lattice of CeNbO_4.25_, reflection conditions: *h0l*: *l*=2n, 0*k0*: *k*=2n, 0*0l*: *l*=2n.

**
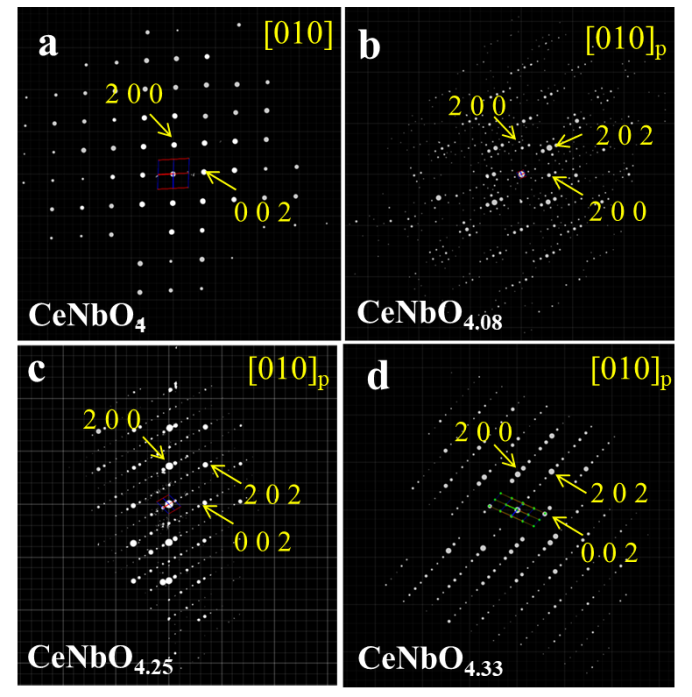
**

**Supplementary Fig. 6** Slices of the reconstructed reciprocal space perpendicular to [010]_p_ that are cut from the reconstructed 3D reciprocal lattice. CeNbO_4_ (**a**), CeNbO_4.08_ (**b**), CeNbO_4.25_ (**c**), and CeNbO_4.33_ (**d**). The strong reflections were the same pseudo-tetragonal symmetry with [010]_p_ zone axis SAED of CeNbO_4.0_.


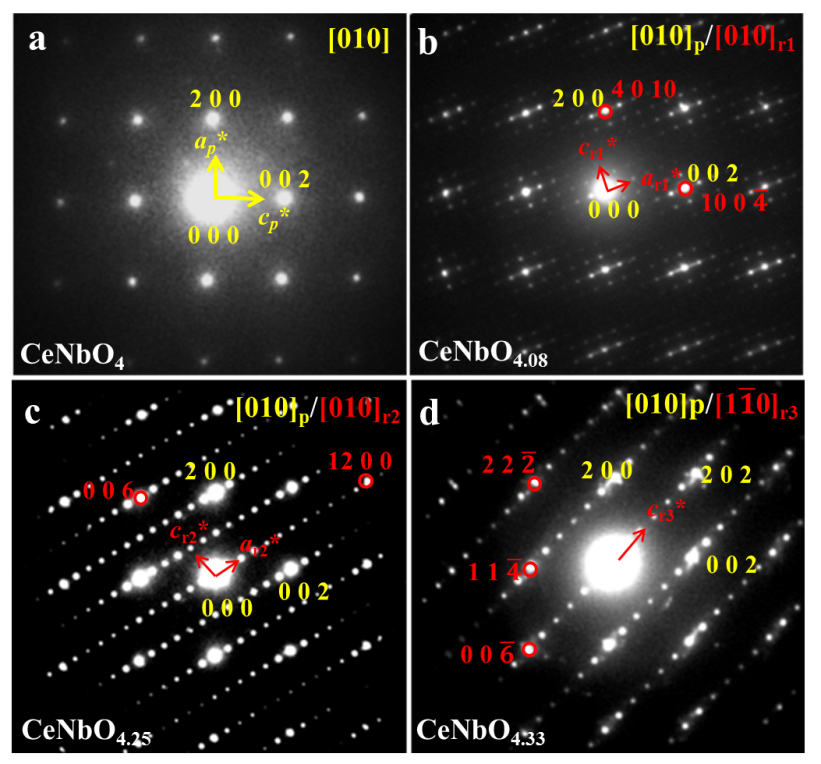


**Supplementary Fig. 7 .**[010]_p_ zone axis SAED of CeNbO_4+δ_ (δ = 0 (**a**), δ = 0.08 (**b**), δ = 0.25 (**c**) and δ = 0.33 (**d**)) phases. Red marks: indexed with super cell, yellow marks: indexed with parent phase.

**
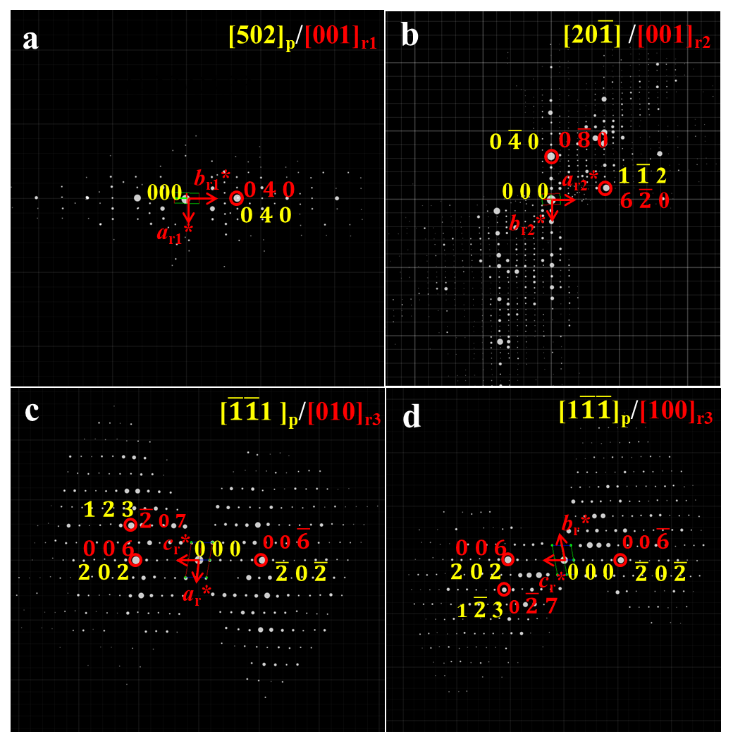
**

**Supplementary Fig. 8** Slices of the reconstructed reciprocal space perpendicular to [001] of CeNbO_4.08_ (**a**) and CeNbO_4.25_ (**b**) that cut from the reconstructed 3D reciprocal lattice. Slices of the reconstructed reciprocal space perpendicular to [010] (**c**) and [100] (**d**) of CeNbO_4.33_. Reds marks: indexed with super cell, yellow marks: indexed with parent phase.


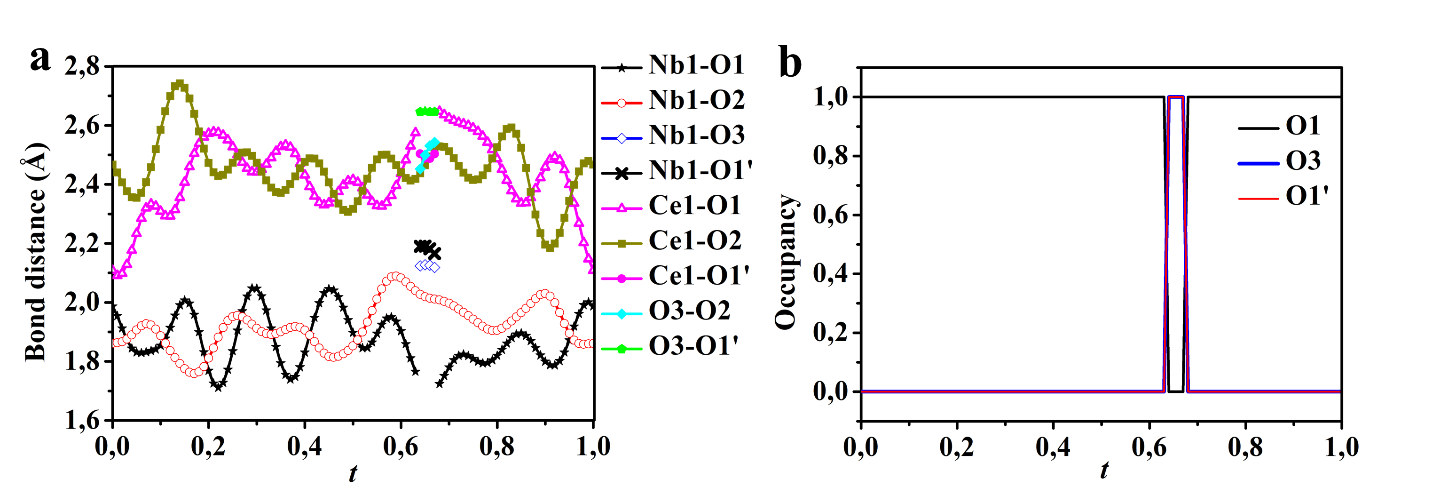


**Supplementary Fig.9 a** The bond lengths in (3+1)D incommensurately modulated structure of CeNbO_4.08_. **b** The occupancy modulation function of O1, O3 and O1.

_
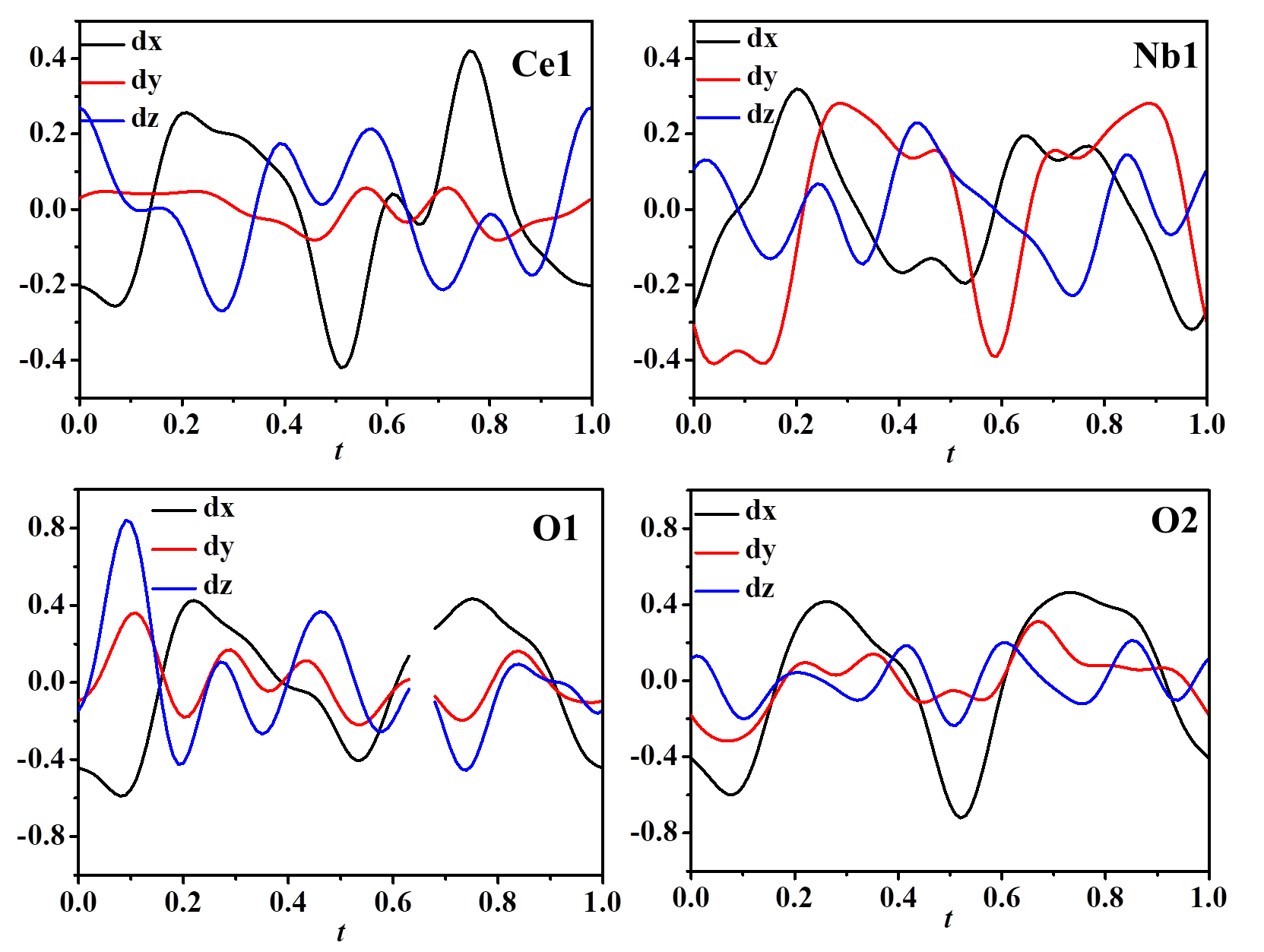
_

**Supplementary Fig. 10** The refined modulation functions of Ce1, Nb1, O1, and O2 in (3+1)D incommensurately modulated structure of CeNbO_4.08_ for 3D ED data (black – x, red – y, blue – z).


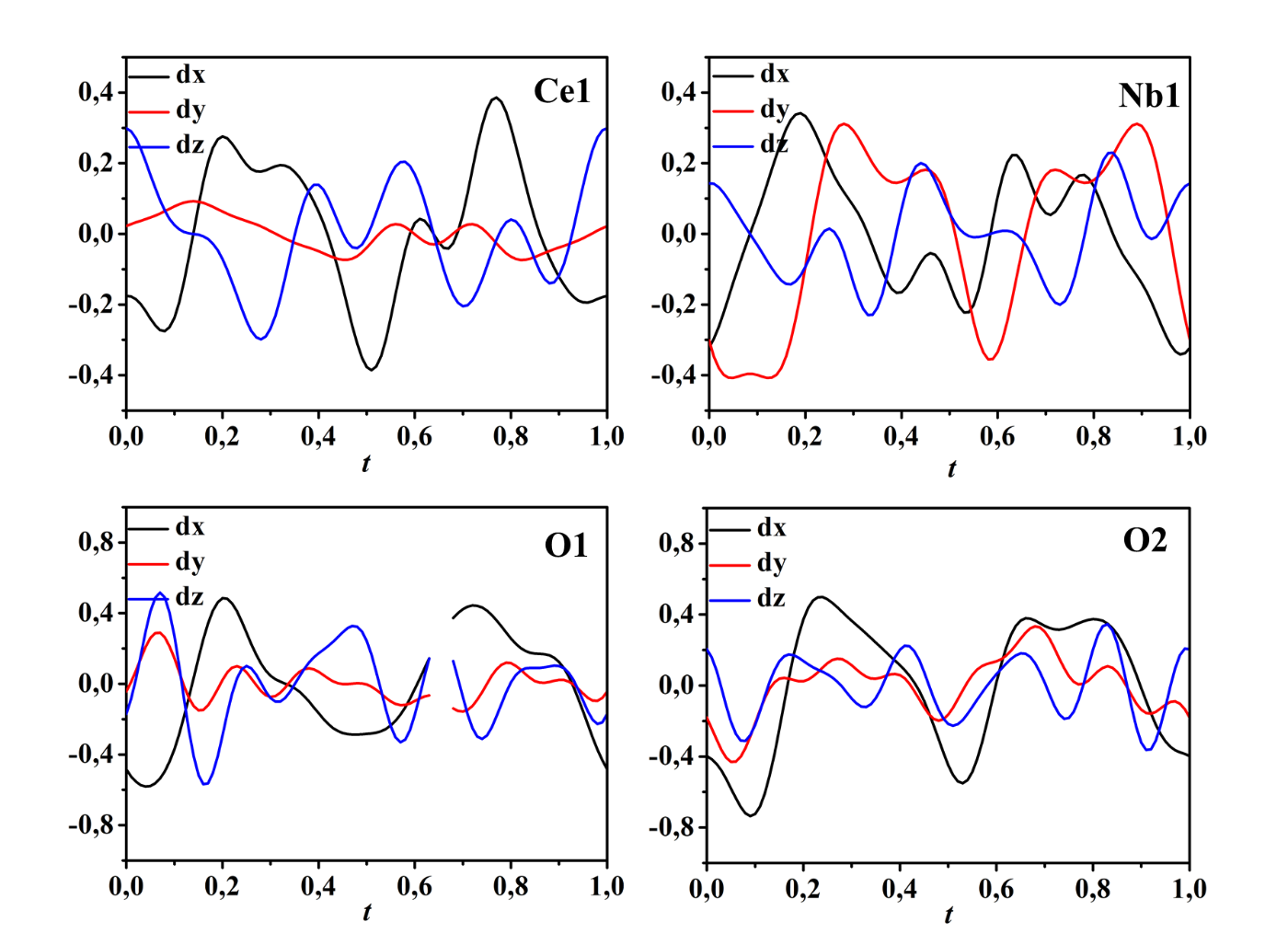


**Supplementary Fig. 11** The refined modulation functions of Ce1, Nb1, O1, and O2 in (3+1)D incommensurately modulated structure of CeNbO_4.08_ for combining SPD and NPD data (black – x, red – y, blue – z).

**
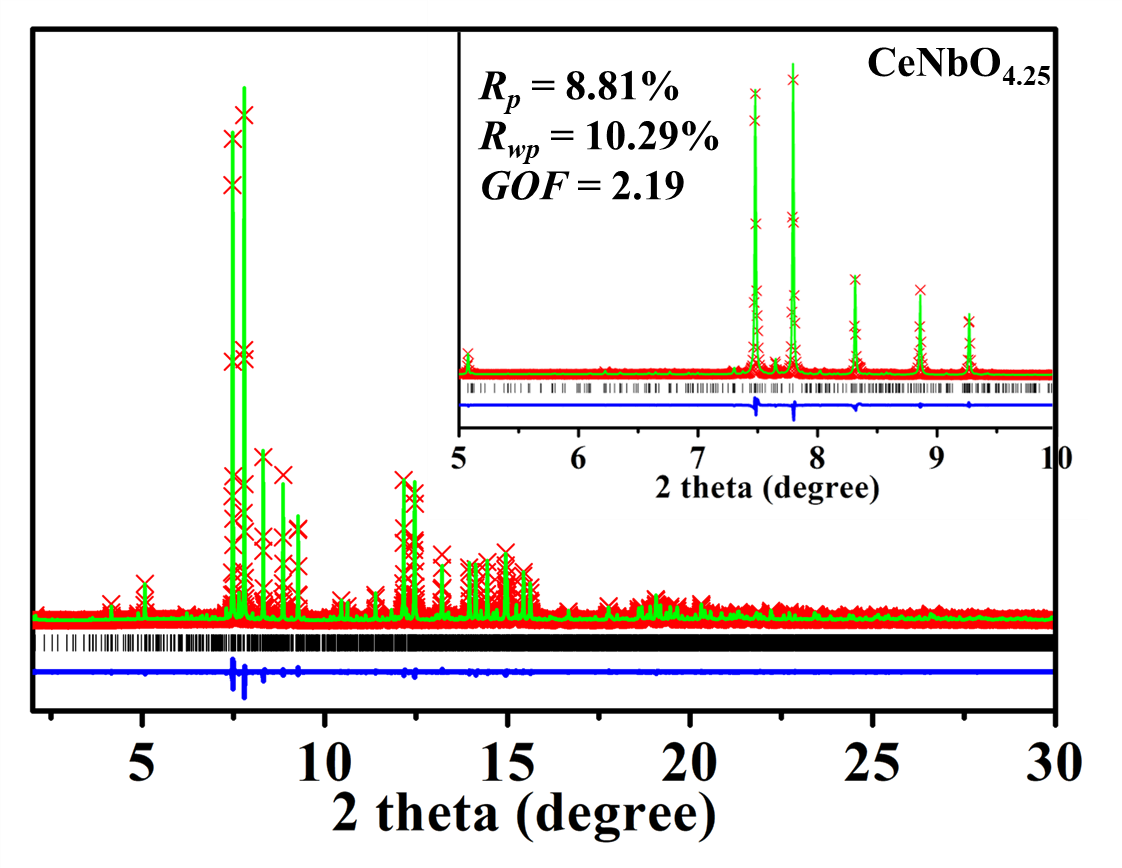
**

**Supplementary Fig. 12** Final Rietveld refinement against the SPD data for CeNbO_4.25_ with supercell structure. (Red × symbol: observed profile, green curve: simulated profile, blue curve: difference profile)


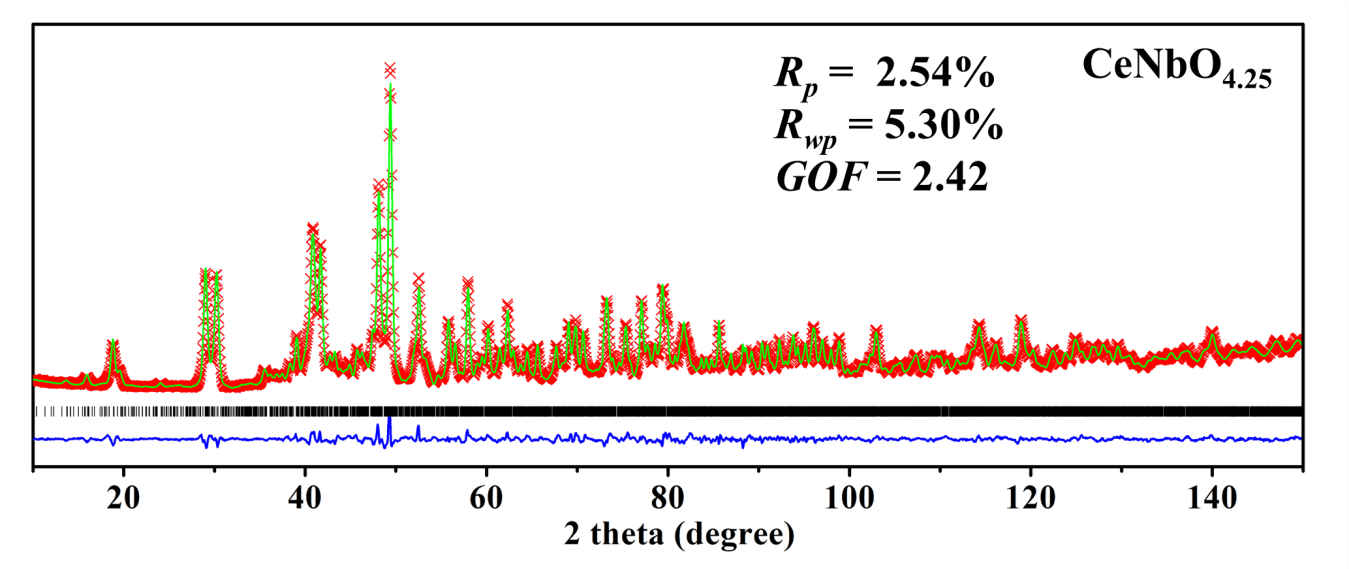


**Supplementary Fig. 13** Final Rietveld refinement against the NPD data for CeNbO_4.25_ with supercell structure. (Red × symbol: observed profile, green curve: simulated profile, blue curve: difference profile)

**
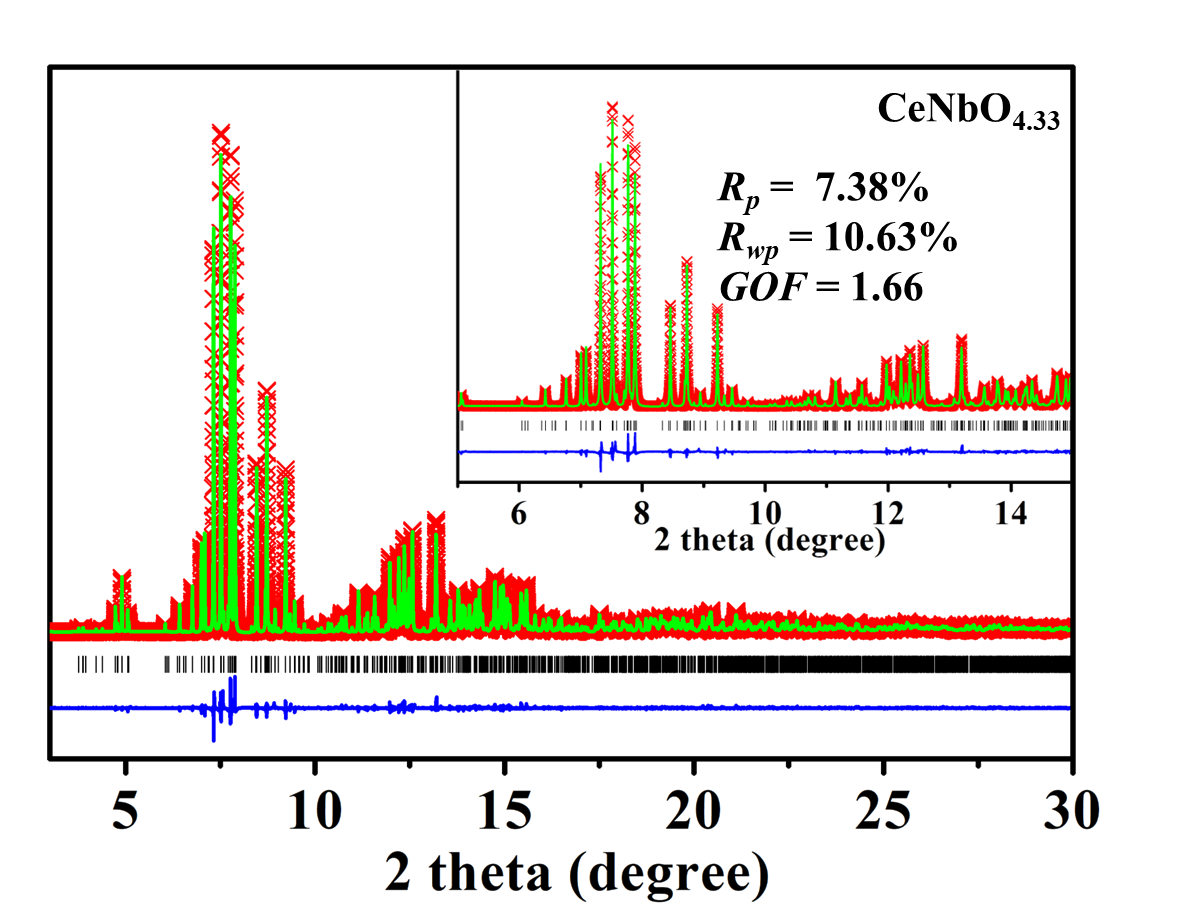
**

**Supplementary Fig. 14** Final Rietveld refinement against the SPD data for CeNbO_4.33_ with supercell structure. (Red × symbol: observed profile, green curve: simulated profile, blue curve: difference profile)

**
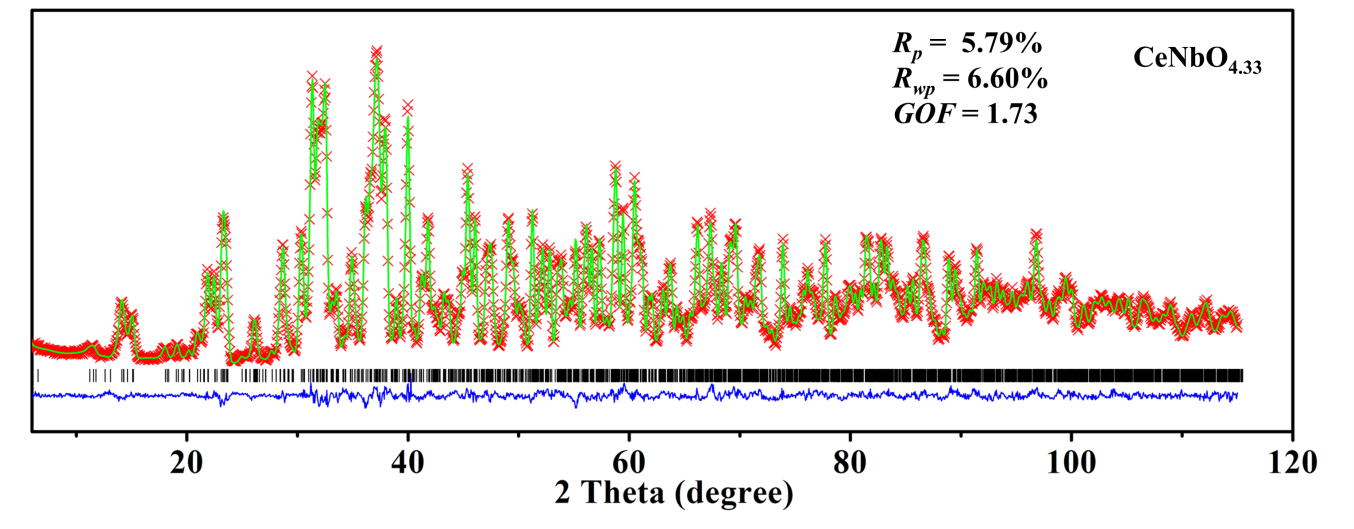
**

**Supplementary Fig. 15** Final Rietveld refinement against the NPD data for CeNbO_4.33_ with supercell structure. (Red × symbol: observed profile, green curve: simulated profile, blue curve: difference profile)

**
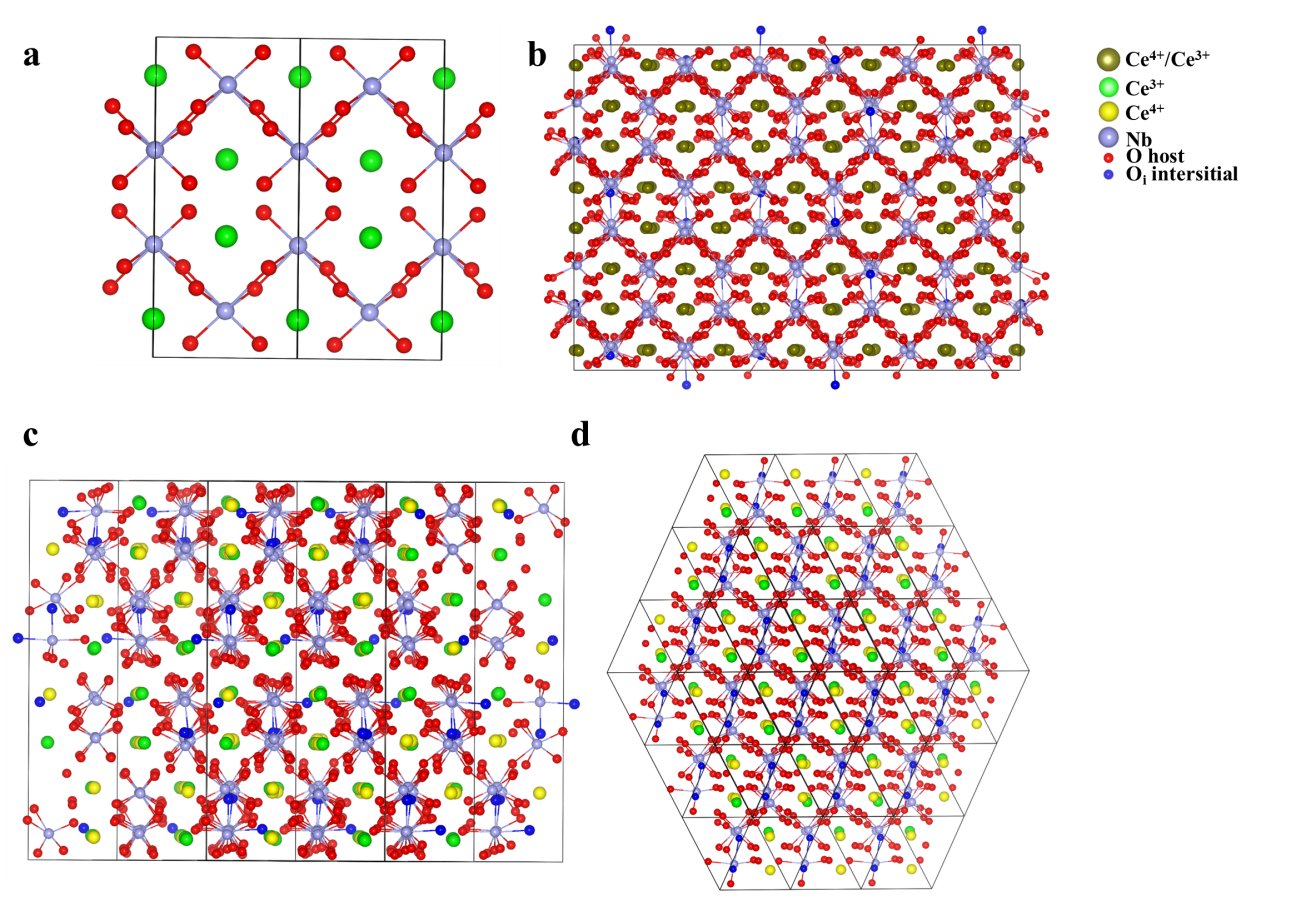
**

**Supplementary Fig. 16** The parent CeNbO_4_ (**a**) and super cells of CeNbO_4.08_ (**b**), CeNbO_4.25_ (**c**), and CeNbO_4.33_ (**d**) projected along the principal axes of [001]_p_. Note that (**b**) was a 6*a* × 2*b* × 6*c* approximant superstructure in (3+1)D incommensurately modulated model along [001]p.


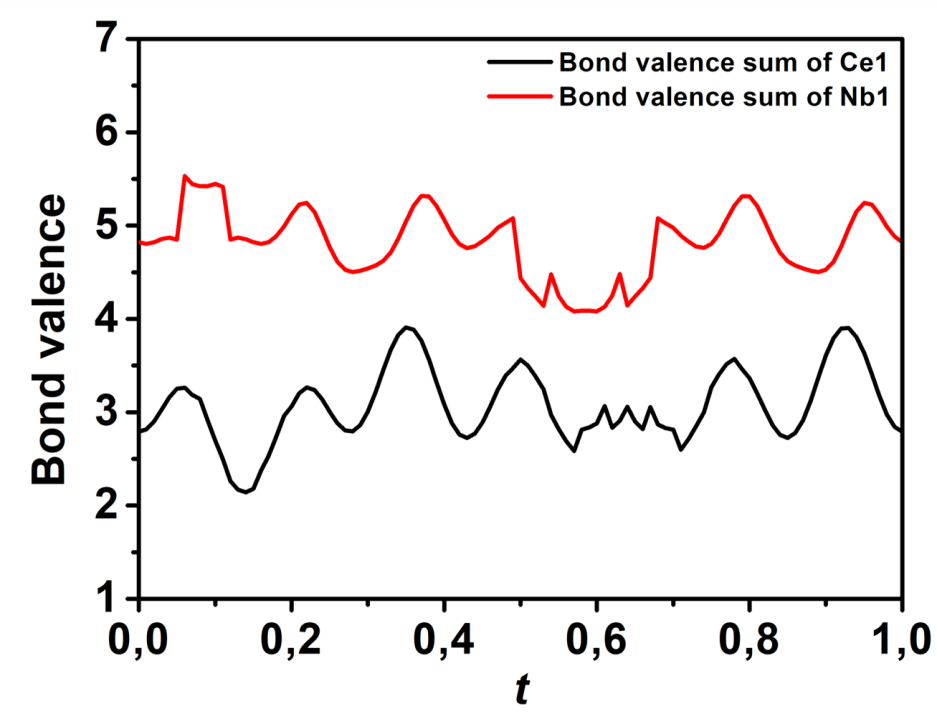


**Supplementary Fig. 17** Bond valence sum of Ce1 (black curve) and Nb1 (red curve) for the (3+1)D incommensurately modulated structure of CeNbO_4.08_. (R_0_ for Ce1 and Nb1 is 2.09 Å and 1.911 Å, respectively. The B constant is 0.37 Å.)^6,7^

**
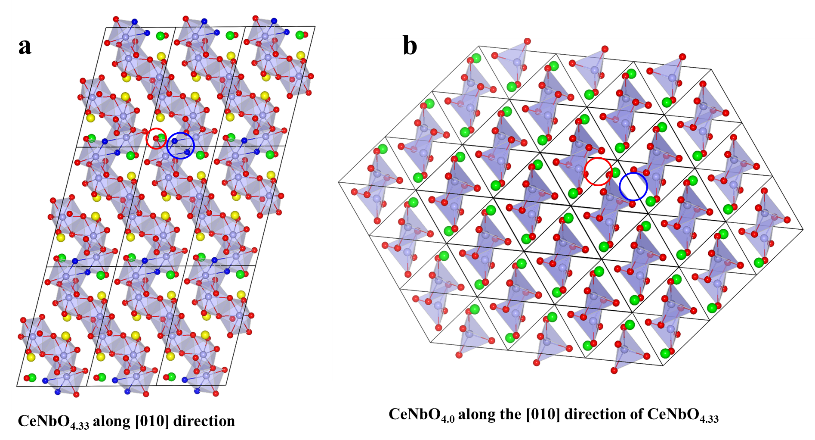
**

**Supplementary Fig. 18 a** CeNbO_4.33_ along the [010] direction. **b** CeNbO_4.0_ along the [010] direction of CeNbO_4.33_. The blue cycle was the position of the interstitial site for O13. The red cycle marks the interstitial site for O11, which was pushed away from the original normal site.

**
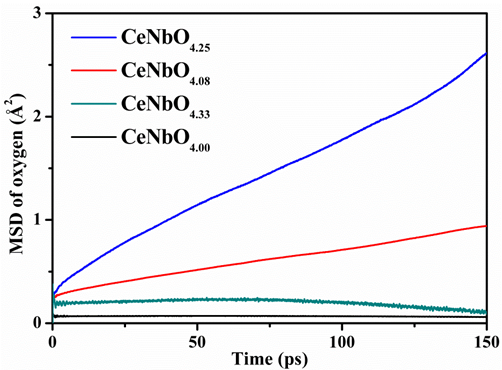
**

**Supplementary Fig.19** Comparison of MSD values of O atoms as a function of simulation time in CeNbO_4+δ_ at 1200℃.


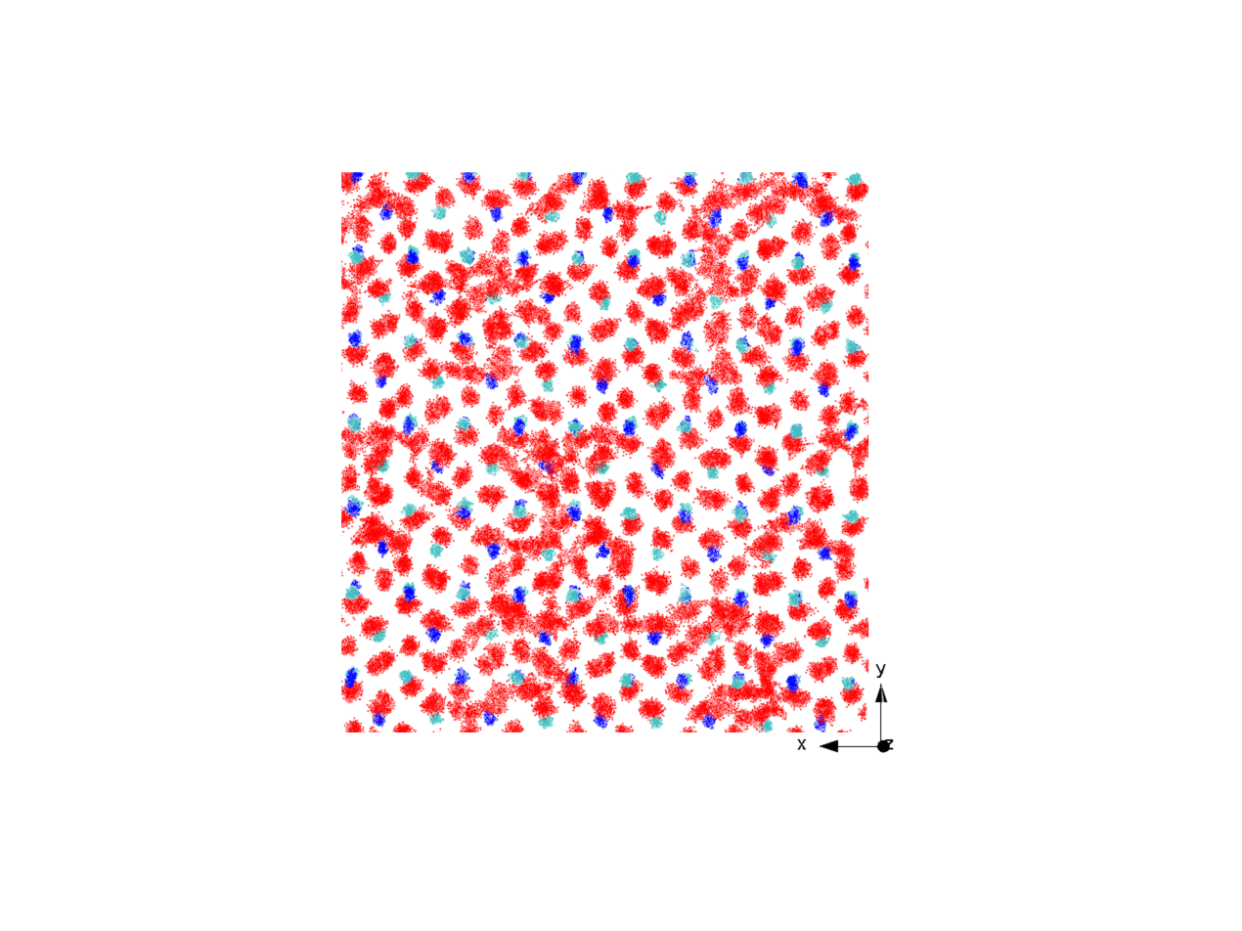


**Supplementary Fig. 20** Trajectory scatter plot for CeNbO_4.25_ at 1200℃ where cyan, blue, and red dots represent Ce^3+/4+^, Nb^5+^, and O^2−^ atoms, respectively. The oxide ion migration displays essentially isotropic feature.

**
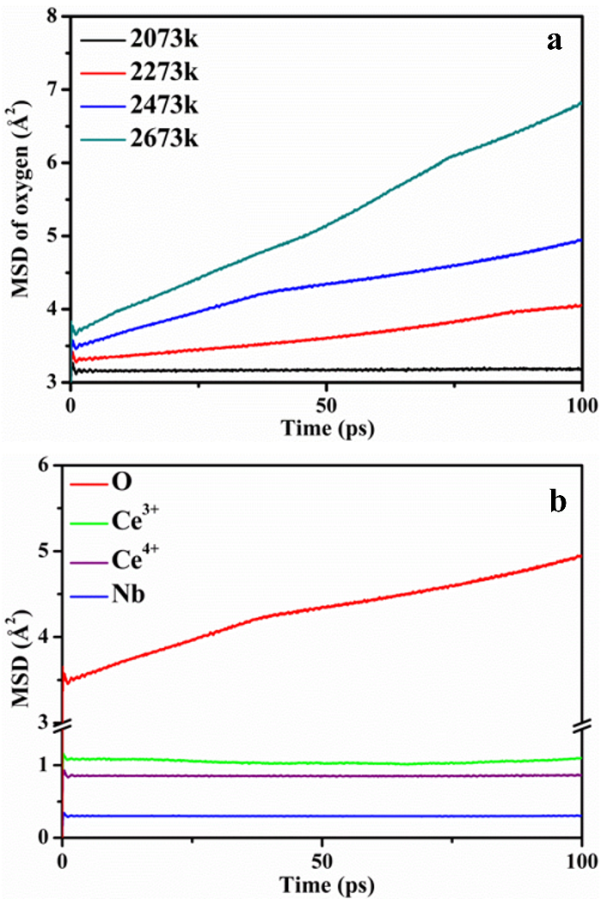
**

**Supplementary Fig. 21** Calculated MSD values for oxygen atom at different temperatures (**a**) and all atoms at 2200 °C (**b**) as function of simulation time for CeNbO_4.33_.


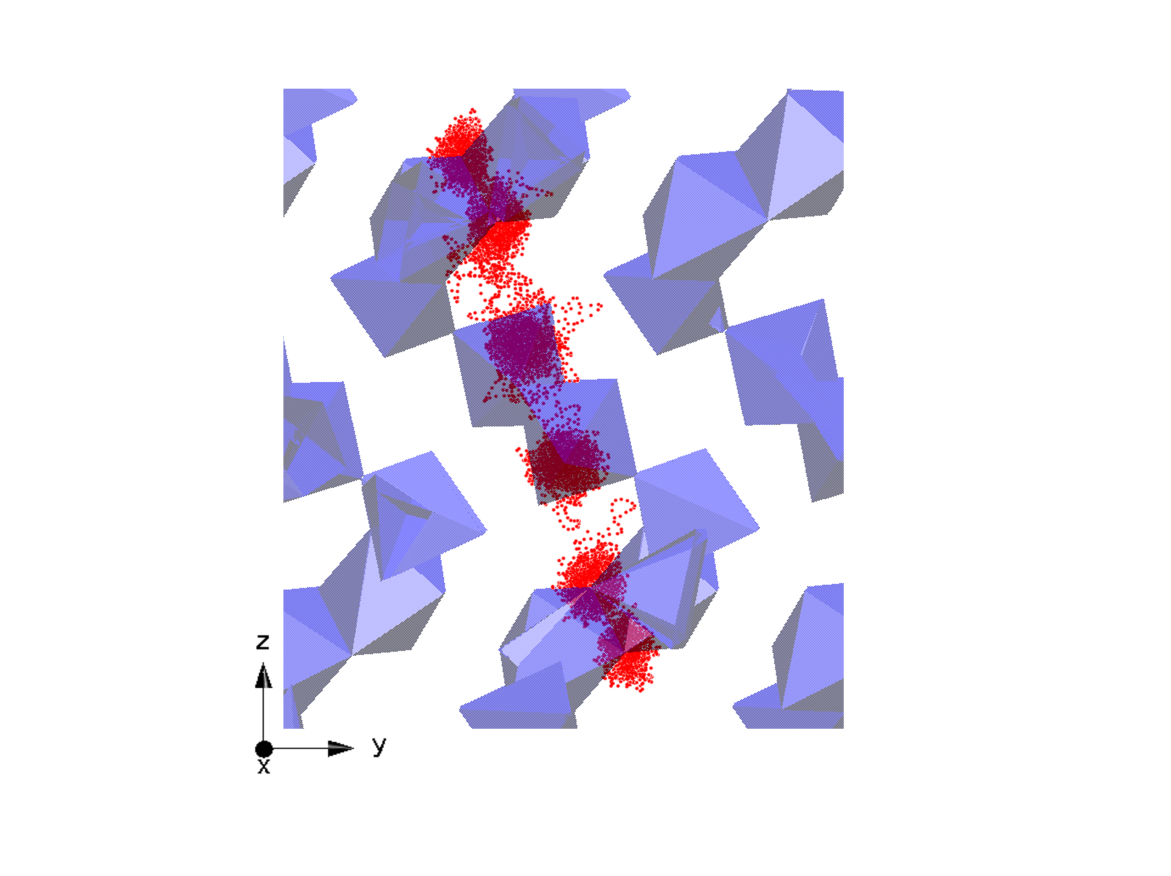


**Supplementary Fig. 22** Trajectory scatter plot of oxide ions showing long migration in CeNbO_4.33_ phase at 2200℃ with the NbO_6_ polyhedral network embedded.


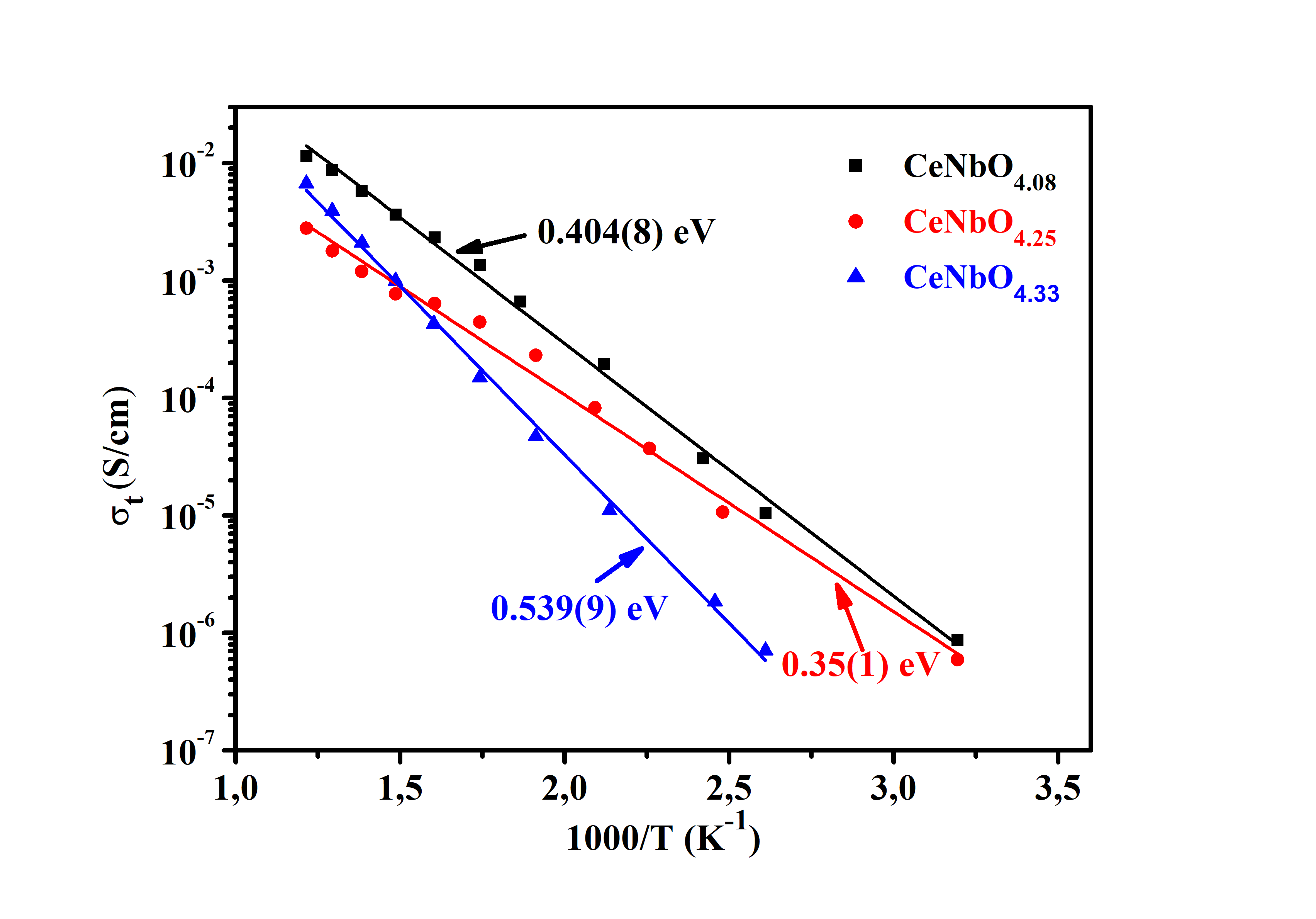


**Supplementary Fig. 23** Arrhenius plot of the conductivities for CeNbO_4+δ_ (δ = 0.08, 0.25, 0.33).

**Supplementary Tables**

**Supplementary Table 1** The details of the 3D ED data collection and processing of CeNbO_4+δ_.

| Fomular | CeNbO_4_ | CeNbO_4.08_ | CeNbO_4.25_ | CeNbO_4.33_ |
| --- | --- | --- | --- | --- |
| Tilt range | -60.98° ~ 59.51° | -56.83° ~ 54.65° | -60.32° ~ 57.39° | -68.30° ~ 58.81° |
| Tilt step | 0.23° | 0.23° | 0.23° | 0.23° |
| Wavelength | 0.0251 Å | 0.0251 Å | 0.0251 Å | 0.0251 Å |
| No. of frames | 468 | 459 | 457 | 496 |
| Data procession | XDS | XDS | XDS | XDS |
| Structure determination | ShelxT/Superflip | ShelxT/Superflip | ShelxT/Superflip | ShelxT/Superflip |
| Crystal system | monoclinic | monoclinic | monoclinic | triclinic |
| Unit cell dimensions | a = 5.42 Å  b = 11.97Å  c = 5.84 Å  β = 95.62° | a = 28.38Å  b = 11.49Å  c = 28.58Å  β = 91.28° | a = 14.74 Å  b = 22.52 Å  c = 11.96 Å  β = 105.56° | a = 6.93 Å  b = 7.18 Å  c = 11.70 Å  α =100.27°  β =100.44°  γ =109.14° |
| space group | *I*2/*a* | *I*2/*a* | *P*2_1_*/c* | *P*$\bar{1}$ |
| Resolution | 0.79 Å | 0.81 Å | 0.80 Å | 0.77 Å |
| Completeness | 64.7% | 79.7% | 86.2% | 69.3% |
| *R_int_* | 0.494 | 0.512 | 0.486 | 0.508 |
| No. of unique reflections | 752 | 7237 | 8599 | 1947 |

**Supplementary Table 2** Selected bond lengths and bond valence sums for CeNbO_4.25_ (for bond lengths <3 Å).

| Ce1^4+^ | Distance | Ce2^4+^ | Distance |
| --- | --- | --- | --- |
| Ce1^4+^-O5 | 2.254(8) | **Ce2^4+^-O4** | 2.254(5) |
| Ce1^4+^-O9 | 2.428(1) | **Ce2^4+^-O33** | 2.338(1) |
| Ce1^4+^-O24 | 2.436(1) | **Ce2^4+^-O35** | 2.230(1) |
| Ce1^4+^-O28 | 2.323(8) | **Ce2^4+^-O48** | 2.374(5) |
| Ce1^4+^-O39 | 2.425(1) | **Ce2^4+^-O30** | 2.722(1) |
| Ce1^4+^-O44 | 2.263(8) | **Ce2^4+^-O23** | 2.322(8) |
| Ce1^4+^-O6 | 2.419(1) | **Ce2^4+^-O47** | 2.582(1) |
| Ce1^4+^-O20 | 2.261(8) | **Ce2^4+^-O51** | 2.321(7) |
|  |  | **Ce2^4+^-O2** | 2.872(6) |
| BVS | 4.05(6) | **BVS** | 3.95(4) |
| Ce3^4+^ | **Distance** | **Ce4^4+^** | **Distance** |
| Ce3^4+^-O1 | 2.635(5) | **Ce4^4+^-O3** | 2.222(8) |
| Ce3^4+^-O18 | 2.551(9) | **Ce4^4+^-O12** | 2.410(1) |
| Ce3^4+^-O27 | 2.342(9) | **Ce4^4+^-O24** | 2.347(9) |
| Ce3^4+^-O45 | 2.687(1) | **Ce4^4+^-O34** | 2.272(1) |
| Ce3^4+^-O46 | 2.434(9) | **Ce4^4+^-O10** | 2.483(1) |
| Ce3^4+^-O49 | 2.306(9) | **Ce4^4+^-O20** | 2.555(1) |
| Ce3^4+^-O7 | 2.558(6) | **Ce4^4+^-O31** | 2.256(8) |
| Ce3^4+^-O25 | 2.374(1) | **Ce4^4+^-O50** | 2.307(9) |
| Ce3^4+^-O36 | 2.357(1) |  |  |
| Ce3^4+^-O43 | 2.549(1) |  |  |
| BVS | 3.71(2) | **BVS** | 4.07(6) |
| Ce5^4+^ | **Distance** | **Ce6^4+^** | **Distance** |
| Ce5^4+^-O13 | 2.326(1) | **Ce6^4+^-O16** | 2.240(9) |
| Ce5^4+^-O25 | 2.435(1) | **Ce6^4+^-O28** | 2.431(1) |
| Ce5^4+^-O43 | 2.235(1) | **Ce6^4+^-O39** | 2.332(8) |
| Ce5^4+^-O15 | 2.357(9) | **Ce6^4+^-O50** | 2.489(1) |
| Ce5^4+^-O23 | 2.428(1) | **Ce6^4+^-O10** | 2.311(9) |
| Ce5^4+^-O35 | 2.752(6) | **Ce6^4+^-O22** | 2.409(1) |
| Ce5^4+^-O41 | 2.533(5) | **Ce6^4+^-O40** | 2.140(9) |
| Ce5^4+^-O11 | 2.362(9) | **Ce6^4+^-O42** | 2.533(1) |
| Ce5^4+^-O19 | 2.421(8) |  |  |
| BVS | 3.85(4) | **BVS** | 4.08(7) |
| Ce7^3+^ | **Distance** | **Ce8^3+^** | **Distance** |
| Ce7^3+^-O21 | 2.680(1) | **Ce8^3+^-O47** | 2.451(8) |
| Ce7^3+^-O26 | 2.458(8) | **Ce8^3+^-O4** | 2.486(1) |
| Ce7^3+^-O36 | 2.412(5) | **Ce8^3+^-O17** | 2.761(1) |
| Ce7^3+^-O38 | 2.496(1) | **Ce8^3+^-O22** | 2.565(7) |
| Ce7^3+^-O2 | 2.770(6) | **Ce8^3+^-O40** | 2.470(1) |
| Ce7^3+^-O45 | 2.516(8) | **Ce8^3+^-O11** | 2.565(1) |
| Ce7^3+^-O8 | 2.526(1) | **Ce8^3+^-O35** | 2.698(7) |
| Ce7^3+^-O29 | 2.449(5) | **Ce8^3+^-O15** | 2.553(1) |
| Ce7^3+^-O1 | 2.458(1) | **Ce8^3+^-O32** | 2.468(6) |
|  |  |  |  |
| BVS | 3.18(2) | **BVS** | 2.96(1) |
| Ce9^3+^ | **Distance** | **Ce10^3+^** | **Distance** |
| Ce9^3+^-O5 | 2.537(1) | **Ce10^3+^-O8** | 2.390(7) |
| Ce9^3+^-O21 | 2.476(9) | **Ce10^3+^-O16** | 2.778(1) |
| Ce9^3+^-O6 | 2.525(7) | **Ce10^3+^-O32** | 2.441(1) |
| Ce9^3+^-O14 | 2.812(1) | **Ce10^3+^-O33** | 2.461(6) |
| Ce9^3+^-O27 | 2.570(1) | **Ce10^3+^-O42** | 2.353(6) |
| Ce9^3+^-O29 | 2.466(1) | **Ce10^3+^-O4** | 2.727(5) |
| Ce9^3+^-O41 | 2.735(5) | **Ce10^3+^-O17** | 2.405(9) |
| Ce9^3+^-O46 | 2.533(1) | **Ce10^3+^-O2** | 2.416(1) |
| Ce9^3+^-O1 | 2.635(5) |  |  |
| Ce9^3+^-O37 | 2.573(7) |  |  |
| BVS | 2.83(1) | **BVS** | 3.28(1) |
| Ce11^3+^ | **Distance** | **Ce12^3+^** | **Distance** |
| Ce11^3+^-O34 | 2.391(8) | **Ce12^3+^-O13** | 2.501(9) |
| Ce11^3+^-O14 | 2.380(1) | **Ce12^3+^-O51** | 2.518(1) |
| Ce11^3+^-O38 | 2.401(8) | **Ce12^3+^-O3** | 2.448(1) |
| Ce11^3+^-O26 | 2.461(1) | **Ce12^3+^-O12** | 2.495(7) |
| Ce11^3+^-O9 | 2.428(8) | **Ce12^3+^-O18** | 2.560(1) |
| Ce11^3+^-O31 | 2.515(1) | **Ce12^3+^-O30** | 2.497(7) |
| Ce11^3+^-O37 | 2.539(1) | **Ce12^3+^-O49** | 2.300(1) |
| Ce11^3+^-O44 | 2.675(1) | **Ce12^3+^-O36** | 2.837(5) |
| Ce11^3+^-O29 | 2.789(5) |  |  |
|  |  |  |  |
| BVS | 3.25(3) | **BVS** | 3.15(1) |
| Nb1^5+^ | **Distance** | **Nb2^5+^** | **Distance** |
| Nb1^5+^-O7 | 2.129(7) | **Nb2^5+^-O15** | 1.829(1) |
| Nb1^5+^-O11 | 2.043(1) | **Nb2^5+^-O23** | 2.462(1) |
| Nb1^5+^-O27 | 2.013(1) | **Nb2^5+^-O24** | 2.417(1) |
| Nb1^5+^-O35 | 1.988(9) | **Nb2^5+^-O28** | 1.907(1) |
| Nb1^5+^-O41 | 2.017(7) | **Nb2^5+^-O47** | 1.900(9) |
| Nb1^5+^-O1 | 1.933(8) | **Nb2^5+^-O10** | 1.910(8) |
| Nb1^5+^-O43 | 2.559(1) |  |  |
| BVS | 4.83(1) | **BVS** | 4.78(5) |
| Nb3^5+^ | **Distance** | **Nb4^5+^** | **Distance** |
| Nb3^5+^-O9 | 1.860(8) | **Nb4^5+^-O5** | 1.887(1) |
| Nb3^5+^-O12 | 1.859(1) | **Nb4^5+^-O13** | 2.342(1) |
| Nb3^5+^-O18 | 2.170(1) | **Nb4^5+^-O25** | 1.921(1) |
| Nb3^5+^-O31 | 1.874(1) | **Nb4^5+^-O37** | 1.896(9) |
| Nb3^5+^-O45 | 1.947(9) | **Nb4^5+^-O18** | 1.937(1) |
| Nb3^5+^-O20 | 2.655(1) | **Nb4^5+^-O27** | 2.266(1) |
| BVS | 4.94(4) | **BVS** | 4.71(2) |
| Nb5^5+^ | **Distance** | **Nb6^5+^** | **Distance** |
| Nb5^5+^-O29 | 1.903(4) | **Nb6^5+^-O24** | 1.966(9) |
| Nb5^5+^-O41 | 2.028(1) | **Nb6^5+^-O47** | 2.278(1) |
| Nb5^5+^-O48 | 1.826(1) | **Nb6^5+^-O51** | 1.833(1) |
| Nb5^5+^-O13 | 2.038(8) | **Nb6^5+^-O17** | 1.925(9) |
| Nb5^5+^-O34 | 1.945(1) | **Nb6^5+^-O38** | 1.875(1) |
| Nb5^5+^-O37 | 2.401(1) | **Nb6^5+^-O44** | 2.338(1) |
| BVS | 4.90(1) | **BVS** | 4.86(4) |
| Nb7^5+^ | **Distance** | **Nb8^5+^** | **Distance** |
| Nb7^5+^-O4 | 2.029(4) | **Nb8^5+^-O3** | 1.913(1) |
| Nb7^5+^-O7 | 1.992(1) | **Nb8^5+^-O23** | 2.088(8) |
| Nb7^5+^-O33 | 1.913(1) | **Nb8^5+^-O42** | 1.874(7) |
| Nb7^5+^-O36 | 1.959(4) | **Nb8^5+^-O19** | 2.030(1) |
| Nb7^5+^-O2 | 1.854(1) | **Nb8^5+^-O30** | 1.844(1) |
| Nb7^5+^-O49 | 2.554(1) | **Nb8^5+^-O10** | 2.695(1) |
| BVS | 4.76(1) | **BVS** | 4.84(3) |
| Nb9^5+^ | **Distance** | **Nb10^5+^** | **Distance** |
| Nb9^5+^-O8 | 1.848(1) | **Nb10^5+^-O21** | 1.924(1) |
| Nb9^5+^-O16 | 2.355(1) | **Nb10^5+^-O39** | 1.913(1) |
| Nb9^5+^-O21 | 2.258(1) | **Nb10^5+^-O50** | 2.477(1) |
| Nb9^5+^-O26 | 1.859(1) | **Nb10^5+^-O20** | 1.955(8) |
| Nb9^5+^-O50 | 1.964(8) | **Nb10^5+^-O45** | 2.141(1) |
| Nb9^5+^-O14 | 1.920(1) | **Nb10^5+^-O46** | 1.851(1) |
| BVS | 4.88(5) | **BVS** | 4.79(4) |
| Nb11^5+^ | **Distance** | **Nb12^5+^** | **Distance** |
| Nb11^5+^-O16 | 1.954(7) | **Nb12^5+^-O40** | 1.959(9) |
| Nb11^5+^-O6 | 1.871(1) | **Nb12^5+^-O11** | 2.195(1) |
| Nb11^5+^-O14 | 2.223(1) | **Nb12^5+^-O19** | 2.023(1) |
| Nb11^5+^-O17 | 2.220(1) | **Nb12^5+^-O49** | 1.955(1) |
| Nb11^5+^-O22 | 1.857(1) | **Nb12^5+^-O32** | 1.901(8) |
| Nb11^5+^-O44 | 1.982(8) | **Nb12^5+^-O43** | 1.949(1) |
| BVS | 4.86(4) | **BVS** | 4.92(1) |

**Supplementary Table 3** Selected bond lengths and bond valence sums for CeNbO_4.33_ (for bond lengths <3 Å).

| Ce1^4+^ | Distance | Ce2^4+^ | Distance |
| --- | --- | --- | --- |
| Ce1^4+^-O11 | 2.220(4) | **Ce2^4+^-O5** | 2.205(5) |
| Ce1^4+^-O6 | 2.261(5) | **Ce2^4+^-O10** | 2.249(5) |
| Ce1^4+^-O3 | 2.335(5) | **Ce2^4+^-O9** | 2.275(4) |
| Ce1^4+^-O10 | 2.349(5) | **Ce2^4+^-O5** | 2.352(5) |
| Ce1^4+^-O4 | 2.360(5) | **Ce2^4+^-O8** | 2.407(5) |
| Ce1^4+^-O12 | 2.401(5) | **Ce2^4+^-O4** | 2.415(5) |
| Ce1^4+^-O1 | 2.505(5) | **Ce2^4+^-O6** | 2.462(4) |
| Ce1^4+^-O13 | 2.595(5) | **Ce2^4+^-O8** | 2.522(5) |
| Ce1^4+^-O9 | 2.669(4) |  |  |
| BVS | 4.04(1) | **BVS** | 3.99(1) |
| Ce3^3+^ | **Distance** |  |  |
| Ce3^3+^-O7 | 2.335(5) |  |  |
| Ce3^3+^-O11 | 2.477(4) |  |  |
| Ce3^3+^-O7 | 2.484(5) |  |  |
| Ce3^3+^-O12 | 2.502(5) |  |  |
| Ce3^3+^-O1 | 2.601(4) |  |  |
| Ce3^3+^-O3 | 2.650(5) |  |  |
| Ce3^3+^-O2 | 2.733(4) |  |  |
| Ce3^3+^-O13 | 2.787(5) |  |  |
| Ce3^3+^-O11 | 2.791(4) |  |  |
| Ce3^3+^-O2 | 2.803(4) |  |  |
| BVS | 3.09(1) |  |  |
| Nb1^5+^ | **Distance** | **Nb2^5+^** | **Distance** |
| Nb1^5+^-O12 | 1.855(5) | **Nb2^5+^-O1** | 1.822(5) |
| Nb1^5+^-O13 | 1.929(5) | **Nb2^5+^-O4** | 1.899(5) |
| Nb1^5+^-O10 | 1.946(4) | **Nb2^5+^-O8** | 1.957(5) |
| Nb1^5+^-O2 | 2.021(4) | **Nb2^5+^-O9** | 1.974(5) |
| Nb1^5+^-O11 | 2.033(4) | **Nb2^5+^-O3** | 2.187(5) |
| Nb1^5+^-O13 | 2.259(5) | **Nb2^5+^-O9** | 2.332(4) |
| BVS | 4.87(2) | **BVS** | 4.82(2) |
| Nb3^5+^ | **Distance** |  |  |
| Nb3^5+^-O6 | 1.889(4) |  |  |
| Nb3^5+^-O2 | 1.901(5) |  |  |
| Nb3^5+^-O7 | 1.901(5) |  |  |
| Nb3^5+^-O3 | 2.047(4) |  |  |
| Nb3^5+^-O5 | 2.077(5) |  |  |
| Nb3^5+^-O8 | 2.236(5) |  |  |
| BVS | 4.86(2) |  |  |

**Supplementary Table 4** Buckingham interatomic potential and shell model parameters used for atomistic simulations of CeNbO_4+_*_δ_*.

| **Interactions** | **A (eV)** | **ρ (Å)** | **C (eV Å^6^)** | **Y (e)** | ***k* (eV Å^−2^)** | **Reference** |
| --- | --- | --- | --- | --- | --- | --- |
| Ce^3+^ − O^2−^ | 2010.18 | 0.3449 | 23.11 | / | / | _8_ |
| Ce^4+^ − O^2−^ | 1017.4 | 0.3949 | 0.0 | / | / | _9_ |
| Nb^5+^ − O^2−^ | 1036.63 | 0.3900 | 0.0 | / | / | _10_ |
| O^2−^ − O^2−^ | 9547.96 | 0.2072 | 32.0 | -2.04 | 6.3 | _11_ |

**Supplementary Table 5** Experimental and calculated cell parameters of CeNbO_4+_*_δ_*.

| **Compound** | **Parameter** | **Experimental** | **Calculated** | **Difference** |
| --- | --- | --- | --- | --- |
| **CeNbO_4.00_** | *a* (Å) | 5.16269 | 5.43723 | 5.32% |
|  | *b* (Å) | 11.4067 | 11.1831 | -1.96% |
|  | *c* (Å) | 5.53779 | 5.43723 | -1.82% |
|  | *β* (°) | 94.6036 | 90.0000 | -4.87% |
|  |  |  |  |  |
| **CeNbO_4.08_** | *a* (Å) | 28.2907 | 29.2097 | 3.25% |
|  | *b* (Å) | 11.4861 | 11.2333 | -2.20% |
|  | *c* (Å) | 28.5415 | 29.1580 | 2.16% |
|  | *β* (°) | 91.4170 | 90.3923 | -1.12% |
|  |  |  |  |  |
| **CeNbO_4.25_** | *a* (Å) | 14.3896 | 15.0062 | 4.29% |
|  | *b* (Å) | 22.8002 | 22.8133 | 0.06% |
|  | *c* (Å) | 11.8390 | 12.0036 | 1.39% |
|  | *β* (°) | 105.077 | 106.546 | 1.40% |
|  |  |  |  |  |
| **CeNbO_4.33_** | *a* (Å) | 6.72592 | 6.58466 | -2.10% |
|  | *b* (Å) | 6.90676 | 7.19789 | 4.22% |
|  | *c* (Å) | 11.2684 | 11.8005 | 4.72% |
|  | *α* (°) | 99.1256 | 101.637 | 2.53% |
|  | *β* (°) | 99.1256 | 97.8638 | -1.27% |
|  | *γ* (°) | 110.457 | 109.608 | -0.77% |

**Supplementary References:**

1. S. Smeets, B. Wang, M. Cichocka, J. Angstrom, W. Wan. Instamatic (Version 0.6) Zenodo, 2018.
2. W. Kabsch. Acta Cryst. 2010, D66, 125–132.
3. Wan, W.; Sun, J.; Su, J.; Hovmoller, S.; Zou, X. Three Dimensional Rotation Electron Diffraction Software RED for Automated Data Collection and Data Processing. J. Appl. Cryst. 2013, 46, 1863-1873.
4. Sheldrick G. M., SHELXT-Integrated space-group and crystal-structure determination, Acta Cryst.,2015, A71, 3-8.
5. Palatinus, L.; Capuis, G. SUPERFLIP– a computer program for the solution of crystal structures by charge flipping in arbitrary dimensions. J. Appl. Crystallogr. 2007, 40, 786-790.
6. Brese and O'Keeffe, (1991), Bond-valence parameters for solids, Acta Cryst. B47, 192-197.
7. Brown and Altermatt, Bond-valence parameters obtained from a systematic analysis of the Inorganic Crystal Structure Database (1985), Acta Cryst. B41, 244-247
8. Minervini, L.; Zacate, M. O.; Grimes, R. W., Defect cluster formation in M_2_O_3_-doped CeO_2_. *Solid State Ionics* **1999,** 116, (3), 339-349.
9. Lewis, G.; Catlow, C., Potential models for ionic oxides. *Journal of Physics C: Solid State Physics* **1985,** 18, (6), 1149.
10. Sepliarsky, M.; Asthagiri, A.; Phillpot, S. R.; Stachiotti, M. G.; Migoni, R. L., Atomic-level simulation of ferroelectricity in oxide materials. *Current Opinion in Solid State & Materials Science* **2005,** 9, (3), 107-113.
11. Chroneos, A.; Parfitt, D.; Kilner, J. A.; Grimes, R. W., Anisotropic oxygen diffusion in tetragonal La_2_NiO_4+δ_: molecular dynamics calculations. *Journal of Materials Chemistry* **2010,** 20, (2), 266-270.
